# Global burden of maternal disorders attributable to malnutrition from 1990 to 2019 and predictions to 2035: worsening or improving?

**DOI:** 10.3389/fnut.2024.1343772

**Published:** 2024-02-15

**Authors:** Tongtong Xu, Chenxian Dong, Jianjiang Shao, Chaojing Huo, Zuhai Chen, Zhengyang Shi, Teng Yao, Chenyang Gu, Wanting Wei, Dongsheng Rui, Xiaoju Li, Yunhua Hu, Jiaolong Ma, Qiang Niu, Yizhong Yan

**Affiliations:** ^1^Department of Preventive Medicine, School of Medicine, Shihezi University, Shihezi, Xinjiang, China; ^2^Key Laboratory for Prevention and Control of Emerging Infectious Diseases and Public Health Security, The Xinjiang Production and Construction Corps, Shihezi, Xinjiang, China; ^3^Key Laboratory of Preventive Medicine, Shihezi University, Shihezi, Xinjiang, China; ^4^Key Laboratory of Xinjiang Endemic and Ethnic Diseases (Ministry of Education), School of Medicine, Shihezi University, Xinjiang, Shihezi, China

**Keywords:** global burden, malnutrition, maternal disorders, prediction, epidemiology

## Abstract

**Background and aims:**

Maternal malnutrition is a major global public health problem that can lead to serious maternal diseases. This study aimed to analyze and predict the spatio-temporal trends in the burden of maternal disorders attributable to malnutrition, and to provide a basis for scientific improvement of maternal malnutrition and targeted prevention of maternal disorders.

**Methods:**

Data on maternal disorders attributable to malnutrition, including number of deaths, disability-adjusted life years (DALYs), population attributable fractions (PAFs), age-standardized mortality rates (ASMRs), and age-standardized DALY rates (ASDRs) were obtained from the Global Burden of Disease Study 2019 to describe their epidemiological characteristics by age, region, year, and type of disease. A log-linear regression model was used to calculate the annual percentage change (AAPC) of ASMR or ASDR to reflect their temporal trends. Bayesian age-period-cohort model was used to predict the number of deaths and mortality rates to 2035.

**Results:**

Global number of deaths and DALYs for maternal disorders attributable to malnutrition declined by 42.35 and 41.61% from 1990 to 2019, with an AAPC of –3.09 (95% CI: −3.31, −2.88) and –2.98 (95% CI: −3.20, −2.77) for ASMR and ASDR, respectively. The burden was higher among younger pregnant women (20–29 years) in low and low-middle socio-demographic index (SDI) regions, whereas it was higher among older pregnant women (30–39 years) in high SDI region. Both ASMR and ASDR showed a significant decreasing trend with increasing SDI. Maternal hemorrhage had the highest burden of all diseases. Global deaths are predicted to decline from 42,350 in 2019 to 38,461 in 2035, with the ASMR declining from 1.08 (95% UI: 0.38, 1.79) to 0.89 (95% UI: 0.47, 1.31).

**Conclusion:**

Maternal malnutrition is improving globally, but in the context of the global food crisis, attention needs to be paid to malnutrition in low SDI regions, especially among young pregnant women, and corresponding measures need to be taken to effectively reduce the burden of disease.

## Introduction

Maternal malnutrition is a major global public health problem. The United Nations International Children’s Emergency Fund estimated that the number of severely malnourished pregnant and lactating women in countries affected by the current food and nutrition crisis increased by 25% between 2020 (5.5 million) and 2022 (6.9 million), with more than 1 billion adolescent girls and women worldwide suffering from malnutrition (1). The Sustainable Development Goals (SDGs) issued by the United Nations General Assembly in 2015 proposed to end all forms of malnutrition and address the nutritional needs of adolescent girls and pregnant women by 2030 (2). Despite concerted efforts, the prevalence of malnutrition remains high, especially in low-and middle-income countries, and progress in reducing malnutrition remains slow (3).

The occurrence of maternal diseases is closely related to malnutrition. Pregnant women need to consume adequate nutrients during pregnancy, including protein, carbohydrate, fat, vitamins and minerals, to maintain their own physiological needs and the growth and development of the fetus (4). If a pregnant woman is chronically malnourished, it will lead to a decrease in immunity, leaving them vulnerable to inflammatory and infectious diseases (5). Iron deficiency in pregnant women can easily induce anemia and is also a high risk factor for postpartum hemorrhage (6). Inadequate thyroid hormone synthesis due to iodine deficiency during pregnancy increases the rate of miscarriage (7). Low vitamin B12 levels can lead to elevated homocysteine levels, which can precipitate adverse outcomes including miscarriage and preeclampsia (8). In addition, a cohort study based on 9,287 women giving birth showed an increased risk of hyperemesis gravidarum and postpartum hemorrhage in overweight women (9). In addition, due to socio-economic, gender bias, and racial discrimination, global inequalities in nutritional problems and maternal illnesses have resulted in more than one-third of 126 low-and middle-income countries being undernourished (10), with WHO estimating that sub-Saharan Africa alone will account for about 70% of global maternal deaths in 2020, with a maternal mortality ratio that is 136 times higher than that of Australia and New Zealand (11). It is clear that malnutrition can cause significant maternal disorders, posing a serious threat to maternal health and life, and is marked by serious inequalities.

Given the rising levels of maternal malnutrition, the serious life-threatening impact of maternal diseases and global inequalities, understanding the spatial–temporal pattern of the burden of maternal diseases caused by malnutrition is critical to developing a targeted global strategy for the prevention and control of maternal diseases, but no relevant studies are currently available. Therefore, this study utilized the latest data from the Global Burden of Disease (GBD) to provide a comprehensive quantitative assessment of the burden and changing patterns of maternal disorders attributable to malnutrition from 1990 to 2019, as well as a prediction of deaths from 2020 to 2035, to provide a scientific basis for maternal dietary interventions and health education, ultimately achieving a reduction in the burden of maternal disease.

## Materials and methods

### Data collection

Data were obtained from the 2019 Global Burden of Disease Database (GBD 2019) published by the Institute for Health Metrics and Evaluation (IHME, query tool^1^), which provides data from 204 countries or territories, 369 diseases, and 87 epidemiologic data on attributable risk factors. The GBD comparative risk assessment framework was used to estimate malnutrition exposure and attributable disease burden. Sources of exposure data included household surveys, various diet and nutrition surveys, and other epidemiological studies, and modeling methods integrated multiple data inputs, used spatiotemporal Gaussian process regression, and drew on information about age, time, and place to produce the best estimates of risk exposure. Estimation of the attributable burden of disease involves determining the relative risk of a disease outcome for a pair of risk exposure-disease outcomes with sufficient evidence of a causal relationship in a randomized controlled trial, prospective cohort study, or case–control study. Population attributable fractions were estimated on the basis of risk exposures to indicators of malnutrition, such as iron, vitamin A, and zinc deficiency, relative risks of outcomes due to exposure, and theoretical minimum risk exposures (the level below which there is no available evidence supporting a relationship with disease outcomes) (12–14).

Global Burden of Disease divides 204 countries or territories into 21 GBD regions and seven super GBD regions based on geographic location (13). In addition, the socio-demographic index (SDI), a new developmental classification closely linked to social development status and population health outcomes, has a value in the range of (0, 1), with higher values indicating a higher degree of development related to health outcomes (12). The 204 countries or territories are classified into five categories based on the lagged distribution of *per capita* income, the average education level of the population aged 15 and above, and the total fertility rate under the age of 25 as low (≤0.454743), low-middle (0.454743–0.607679), middle (0.607679–0.689504), high-middle (0.689504–0.805129), and high (>0.805129) SDI regions, respectively (15).

All available data from the GBD database on the global burden of the following 10 maternal diseases attributable to malnutrition were used in this study: maternal hemorrhage, maternal sepsis and other maternal infections, maternal hypertensive disorders, maternal obstructed labor and uterine rupture, maternal abortion and miscarriage (MAM), ectopic pregnancy, indirect maternal deaths (defined as deaths caused by existing diseases exacerbated by pregnancy, examples include maternal infections and parasitic diseases during pregnancy, labor and puerperium, and diabetes during pregnancy, labor, and puerperium), late maternal deaths (defined as deaths occurring between 6 weeks and 1 year after the end of pregnancy, excluding accidental deaths), maternal deaths aggravated by HIV/AIDS and other maternal disorders.

### Data analysis

The number of deaths and disability-adjusted life years (DALYs), mortality rates, DALY rates, age-standardized mortality rate (ASMR), age-standardized DALY rate (ASDR), population attributable fraction (PAF), and their 95% uncertainty intervals (95% UIs) were used to analyze the global burden of maternal disorders attributable to malnutrition from 1990 to 2019. The above indicators were obtained from GBD 2019, and their calculation methods have been reported in previous studies (12).

Joinpoint models are a group of linear statistical models widely used to assess trends in the burden of disease over time. It includes both linear regression models and log-linear regression models, the latter of which are commonly used to analyze population-based mortality trends (16). Therefore, a log-linear regression model was used in this study. The model establishes a segmented regression based on the temporal characteristics of the disease distribution, splits the study time into different intervals through a number of connecting points, and optimizes the trend fitting for each interval. The regression coefficients for each interval were weighted to obtain the average annual percentage change (AAPC) and its 95% confidence interval (95% CI) to estimate the overall change over the study period (17, 18), as follows:


$$\ln\left(\gamma \right)=\alpha +\beta_{i}\chi +\varepsilon$$

$$APCs=100\times \left(\exp\left(\beta_{i}\right)-1\right)$$

$$AAPCs=\left\{\exp\left(\sum \omega_{i}\beta_{i}/\sum \omega_{i}\right)-1\right\}\times 100$$

Where γ is ASMR or ASDR, χ is the year, α is the intercept, $\beta_{i}$ is the slope coefficient of each time period, $\omega_{i}$ is the number of years in each time period.

ASMR or ASDR was considered to be increasing if the lower limit of the 95% CI of the AAPC was >0, decreasing if the upper limit was <0, and considered stable if the 95% CI contained 0. In addition, to explore the impact of SDI on the burden of maternal disorders attributable to malnutrition, correlations were assessed at the country or territory level using scatterplots and Pearson correlation analysis.

For disease burden prediction, the Bayesian age-period-cohort (BAPC) model has a low error rate (19) and high coverage (20). Therefore, the BAPC model was used in this study, based on the death data of each age group from 1990 to 2019 in the GBD database, combined with demographic data from 1990 to 2035 provided by IHME^2^, to predict the number of deaths and ASMR of maternal disorders attributable to malnutrition from 2020 to 2035. The BAPC model assumes that temporally adjacent age, period and cohort have similar effects, and uses a second-order random walk to smooth the priors of the three effects. The prior information of the unknown parameters and the sample information are estimated to obtain a posterior distribution, based on which the unknown parameter is inferred (21). In addition, to compare the predictions, we used the mortality rate in 2019 as the baseline, with a 1% increase in deaths per year as a pessimistic reference and a 1% decrease in deaths per year as an optimistic one. The BAPC model with integrated nested Laplace approximation is implemented by using the BAPC package and the INLA package in R.

All statistical analyses were performed using R version 4.3.0^3^ and two-sided *P-*values less than 0.05 were considered statistically significant.

## Results

### PAFs of maternal disorders attributable to malnutrition

The global age-standardized death-PAF of maternal disorders attributable to malnutrition decreased from 24.19% (95% UI: 8.96, 38.47%) in 1990 to 21.56% (95% UI: 7.92, 34.51%) in 2019, with an AAPC of-0.39 (95% CI: −0.44, −0.35) (Table 1), age-standardized DALY-PAF decreased from 24.22% (95% UI: 8.97, 38.44%) in 1990 to 21.52% (95% UI: 7.93, 34.45%) in 2019, with an AAPC of-0.41 (95% CI: −0.45, −0.36) (Supplementary Table S1).

**Table 1:** Global deaths of maternal disorders attributable to maternal malnutrition in 1990 and 2019, and the temporal trend from 1990 to 2019.

**Characteristics**	**1990**			**2019**			**1990–2019**	
	**Deaths No. × 10** ^**3**^ **(95%UI)**	**ASMR per 100,000 (95%UI)**	**Age- standardized PAF, % (95%UI)**	**Deaths No. × 10** ^**3**^ **(95%UI)**	**ASMR per 100,000 (95%UI)**	**Age- standardized PAF, % (95%UI)**	**AAPC of ASMR (95%CI)**	**AAPC of Age- standardized PAF(95%CI)**
**Overall**	73.46 (27.23, 117.53)	2.69 (1.00, 4.31)	24.19 (8.96, 38.47)	42.35 (15.00, 70.28)	1.08 (0.38, 1.79)	21.56 (7.92, 34.51)	–3.09 (−3.31, −2.88)	–0.39 (−0.44, −0.35)
**Disease type**								
Maternal hemorrhage	23.57 (8.65, 38.13)	0.86 (0.32, 1.39)	24.68 (9.14, 39.32)	10.18 (3.63, 17.04)	0.26 (0.09, 0.44)	21.91 (8.01, 35.14)	−4.06 (−4.37, −3.75)	−0.41 (−0.47, −0.35)
Maternal sepsis and other maternal infections	9.54 (3.62, 15.50)	0.35 (0.13, 0.56)	24.96 (9.26, 39.58)	3.75 (1.34, 6.29)	0.10 (0.03, 0.16)	22.26 (8.19, 35.55)	−4.33 (−4.46, −4.20)	−0.39 (−0.42, −0.37)
Maternal hypertensive disorders	9.39 (3.45, 15.21)	0.34 (0.12, 0.54)	23.55 (8.67, 37.57)	5.84 (2.10, 9.73)	0.15 (0.05, 0.25)	21.00 (7.70, 33.86)	−2.73 (−2.84, −2.62)	−0.39 (−0.44, −0.35)
Maternal obstructed labor and uterine rupture	2.06 (0.78, 3.39)	0.08 (0.03, 0.12)	24.51 (9.07, 38.87)	2.30 (0.81, 3.86)	0.06 (0.02, 0.10)	21.80 (8.02, 35.08)	−0.96 (−1.29, −0.62)	−0.41 (−0.44, −0.38)
Maternal abortion and miscarriage	14.29 (5.30, 23.37)	0.54 (0.20, 0.88)	23.93 (8.74, 38.07)	4.38 (1.56, 7.35)	0.11 (0.04, 0.19)	22.42 (8.27, 35.79)	−5.28 (−5.40, −5.15)	−0.23 (−0.25, −0.20)
Ectopic pregnancy	1.37 (0.51, 2.24)	0.05 (0.02, 0.08)	23.85 (8.76, 38.06)	1.41 (0.50, 2.37)	0.04 (0.01, 0.06)	21.89 (7.99, 35.06)	−1.23 (−1.47, −0.98)	−0.29 (−0.33, −0.26)
Indirect maternal deaths	5.13 (1.87, 8.39)	0.18 (0.07, 0.30)	23.97 (8.90, 38.24)	5.25 (1.84, 8.70)	0.13 (0.05, 0.22)	20.82 (7.64, 33.17)	−1.01 (−1.39, −0.63)	−0.49 (−0.54, −0.43)
Late maternal deaths	2.37 (0.86, 3.89)	0.09 (0.03, 0.14)	23.85 (8.90, 37.85)	2.84 (0.98, 4.91)	0.07 (0.02, 0.13)	21.51 (7.92, 34.36)	−0.63 (−0.76, −0.51)	−0.36 (−0.38, −0.33)
Maternal deaths aggravated by HIV/AIDS	0.18 (0.06, 0.32)	0.01 (0.00, 0.01)	24.92 (9.30, 39.43)	0.35 (0.12, 0.64)	0.01 (0.00, 0.02)	22.04 (8.07, 35.31)	0.91 (0.76, 1.06)	−0.42 (−0.44, −0.40)
Other maternal disorders	5.56 (2.07, 8.98)	0.20 (0.08, 0.33)	22.96 (8.45, 36.51)	6.05 (2.09, 10.02)	0.15 (0.05, 0.26)	21.04 (7.67, 33.66)	−0.95 (−1.16, −0.73)	−0.30 (−0.37, −0.23)
**SDI region**								
High SDI	0.23 (0.08, 0.38)	0.05 (0.02, 0.09)	12.57 (4.45, 20.55)	0.18 (0.06, 0.29)	0.04 (0.01, 0.06)	11.23 (4.06, 18.41)	−1.27 (−1.95, −0.58)	−0.39 (−0.42, −0.35)
High-middle SDI	2.99 (1.08, 4.98)	0.48 (0.17, 0.80)	19.37 (6.94, 31.48)	0.70 (0.25, 1.15)	0.10 (0.04, 0.16)	15.90 (5.73, 25.72)	−5.43 (−5.76, −5.10)	−0.69 (−0.86, −0.52)
Middle SDI	13.03 (4.76, 21.48)	1.44 (0.53, 2.37)	19.90 (7.19, 32.24)	4.42 (1.57, 7.34)	0.35 (0.13, 0.59)	18.16 (6.60, 29.36)	−4.69 (−5.15, −4.23)	−0.32 (−0.40, −0.23)
Low-middle SDI	32.43 (11.73, 52.08)	5.95 (2.15, 9.57)	25.35 (9.50, 40.28)	14.46 (5.14, 23.90)	1.54 (0.55, 2.54)	21.17 (7.72, 33.87)	−4.53 (−4.80, −4.26)	−0.63 (−0.69, −0.56)
Low SDI	24.75 (9.37, 40.24)	10.97 (4.14, 17.92)	26.45 (9.89, 41.73)	22.55 (8.06, 37.91)	4.33 (1.55, 7.28)	22.91 (8.44, 36.71)	−3.16 (−3.26, −3.06)	−0.49 (−0.51, −0.47)
**GBD Region**								
High-income Asia Pacific	0.05 (0.02, 0.08)	0.06 (0.02, 0.09)	15.54 (5.50, 25.38)	0.01 (0.00, 0.02)	0.01 (0.00, 0.02)	12.59 (4.51, 20.77)	−4.89 (−5.58, −4.18)	−0.70 (−0.77, −0.64)
High-income North America	0.06 (0.02, 0.10)	0.04 (0.01, 0.07)	11.02 (3.94, 18.07)	0.12 (0.04, 0.19)	0.07 (0.02, 0.11)	11.40 (4.11, 18.81)	1.77 (0.31, 3.26)	0.12 (0.08, 0.15)
Western Europe	0.05 (0.02, 0.08)	0.02 (0.01, 0.04)	9.71 (3.49, 15.82)	0.02 (0.01, 0.03)	0.01 (0.00, 0.02)	8.75 (3.17, 14.23)	−3.09 (−3.93, −2.24)	−0.36 (−0.41, −0.31)
Australasia	0.00 (0.00, 0.00)	0.03 (0.01, 0.05)	11.55 (4.24, 19.02)	0.00 (0.00, 0.00)	0.01 (0.01, 0.02)	10.58 (3.86, 17.56)	−2.08 (−2.73, −1.43)	−0.30 (−0.34, −0.27)
Southern Latin America	0.08 (0.03, 0.13)	0.32 (0.12, 0.52)	13.30 (4.78, 21.47)	0.05 (0.02, 0.08)	0.15 (0.05, 0.24)	11.71 (4.31, 19.20)	−2.81 (−3.26, −2.36)	−0.44 (−0.48, −0.40)
Andean Latin America	0.56 (0.19, 0.91)	3.03 (1.06, 4.98)	21.27 (7.65, 34.62)	0.20 (0.07, 0.35)	0.59 (0.20, 1.05)	15.21 (5.44, 24.98)	−5.49 (−6.10, −4.88)	−1.16 (−1.22, −1.10)
Tropical Latin America	0.78 (0.28, 1.31)	0.98 (0.35, 1.64)	19.60 (6.91, 32.90)	0.34 (0.12, 0.58)	0.28 (0.10, 0.48)	16.78 (5.97, 28.01)	−3.91 (−4.44, −3.37)	−0.53 (−0.55, −0.50)
Central Latin America	0.74 (0.27, 1.21)	0.88 (0.31, 1.44)	15.95 (5.74, 26.21)	0.39 (0.13, 0.65)	0.28 (0.10, 0.48)	13.19 (4.72, 21.60)	−3.72 (−4.26, −3.18)	−0.65 (−0.72, −0.58)
Caribbean	0.42 (0.15, 0.70)	2.25 (0.83, 3.81)	24.16 (8.96, 38.76)	0.43 (0.14, 0.74)	1.75 (0.58, 3.04)	21.17 (7.67, 34.46)	−0.87 (−1.16, −0.58)	−0.45 (−0.50, −0.40)
Eastern Europe	0.17 (0.06, 0.28)	0.15 (0.05, 0.25)	13.10 (4.66, 21.43)	0.04 (0.01, 0.06)	0.04 (0.01, 0.06)	11.70 (4.22, 19.32)	−4.98 (−6.84, −3.08)	−0.40 (−0.46, −0.35)
Central Europe	0.07 (0.02, 0.11)	0.11 (0.04, 0.19)	14.33 (5.05, 23.37)	0.01 (0.00, 0.02)	0.02 (0.01, 0.03)	12.43 (4.48, 20.41)	−6.04 (−7.49, −4.56)	−0.49 (−0.55, −0.42)
Central Asia	0.20 (0.07, 0.33)	0.58 (0.21, 0.94)	20.05 (7.17, 32.47)	0.10 (0.03, 0.16)	0.19 (0.07, 0.32)	17.72 (6.48, 28.87)	−3.78 (−4.69, −2.87)	−0.43 (−0.46, −0.40)
North Africa and Middle East	3.66 (1.33, 6.02)	2.43 (0.89, 3.96)	20.67 (7.56, 33.06)	2.19 (0.78, 3.82)	0.68 (0.24, 1.18)	19.03 (6.99, 30.71)	−4.30 (−4.44, −4.16)	−0.28 (−0.31, −0.24)
South Asia	38.83 (14.22, 62.36)	7.33 (2.69, 11.78)	27.30 (10.31, 43.25)	15.16 (5.39, 25.50)	1.55 (0.55, 2.61)	22.76 (8.33, 36.39)	−5.22 (−5.56, −4.88)	−0.63 (−0.67, −0.59)
Southeast Asia	5.26 (1.94, 8.75)	2.23 (0.82, 3.70)	18.50 (6.57, 29.78)	1.63 (0.59, 2.75)	0.45 (0.16, 0.76)	15.50 (5.59, 25.29)	−5.36 (−5.51, −5.22)	−0.61 (−0.63, −0.58)
East Asia	3.51 (1.24, 6.17)	0.51 (0.18, 0.89)	18.98 (6.86, 31.98)	0.24 (0.08, 0.42)	0.03 (0.01, 0.06)	13.54 (4.84, 22.32)	−8.91 (−10.13, −7.67)	−1.16 (−1.18, −1.13)
Oceania	0.15 (0.06, 0.26)	4.92 (1.85, 8.40)	24.74 (8.92, 39.87)	0.20 (0.07, 0.36)	3.02 (1.06, 5.37)	22.33 (8.03, 36.31)	−1.69 (−1.88, −1.50)	−0.35 (−0.37, −0.34)
Western Sub-Saharan Africa	7.03 (2.58, 11.56)	8.79 (3.23, 14.59)	23.13 (8.56, 36.94)	10.82 (3.68, 18.38)	5.10 (1.74, 8.71)	23.58 (8.75, 37.38)	−1.85 (−1.92, −1.78)	0.07 (0.05, 0.09)
Eastern Sub-Saharan Africa	8.65 (3.20, 14.40)	11.12 (4.08, 18.53)	24.40 (8.96, 38.81)	6.83 (2.44, 11.57)	3.64 (1.30, 6.15)	21.08 (7.70, 33.92)	−3.79 (−3.92, −3.65)	−0.50 (−0.52, −0.48)
Central Sub-Saharan Africa	2.41 (0.91, 4.13)	10.56 (3.96, 18.23)	27.90 (10.80, 43.61)	2.96 (1.04, 5.05)	5.11 (1.80, 8.81)	21.66 (7.88, 35.10)	−2.52 (−2.79, −2.26)	−0.87 (−0.92, −0.83)
Southern Sub-Saharan Africa	0.77 (0.28, 1.28)	2.93 (1.04, 4.87)	21.43 (7.69, 34.97)	0.61 (0.22, 1.05)	1.40 (0.50, 2.39)	20.64 (7.45, 33.68)	−2.46 (−3.97, −0.93)	−0.14 (−0.20, −0.07)

ASMR, age-standardized mortality rate; PAF, population attributable fraction; AAPC, average annual percentage change; UI, uncertainty interval; CI, confidence interval; SDI, socio-demographic index; GBD, Global Burden of Disease Study.

**Figure 1:**
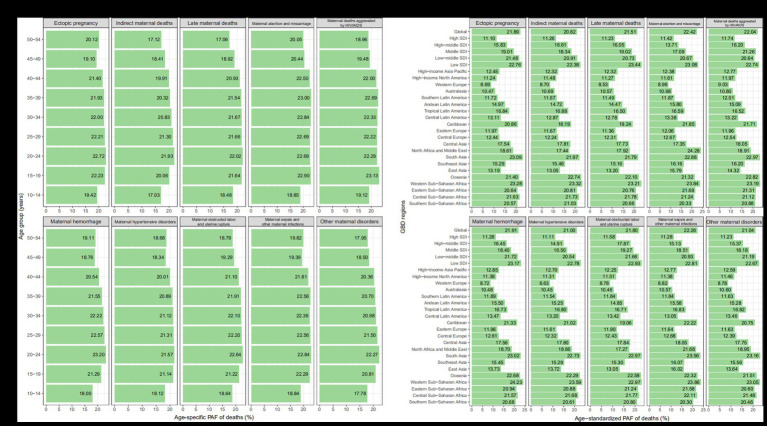
PAF of specific GBD maternal disorders in ASMR attributable to maternal malnutrition by age group and by region in 2019. **(A)** By age group. **(B)** By region. ASMR, age-standardized mortality rate; GBD, Global Burden of Disease Study; SDI, socio-demographic index.

With age, death-PAFs showed an increasing and then decreasing trend, with lower PAFs in the 10–14, 45–49, and 50–54 age groups (Figure 1A); a similar pattern was observed for DALY-PAFs (Supplementary Figure S1A).

For different regions, except for maternal deaths aggravated by HIV/AIDS, the death-PAFs for all diseases showed a downward trend from low to high SDI, with the highest in Western Sub-Saharan Africa and the lowest in Western Europe (Table 1 and Figure 1B); a similar pattern was observed for DALY-PAFs (Supplementary Table S1 and Supplementary Figure S1B). From 1990 to 2019, death-PAFs and DALY-PAFs decreased in all SDI regions, with the fastest declines observed in the high-middle SDI region (Table 1 and Supplementary Table S1).

Among the 10 maternal diseases attributable to malnutrition, death-PAFs were highest for maternal miscarriage and lowest for indirect maternal death (Table 1); the same was true for DALY-PAFs (Supplementary Table S1). From 1990 to 2019, both death-PAFs and DALY-PAFs for each disease decreased, with the fastest decline observed for maternal indirect deaths (Table 1 and Supplementary Table S1).

### Global burden of maternal disorders attributable to malnutrition in 2019

Globally, the number of maternal deaths attributable to malnutrition was 42,350 (95% UI: 15,000, 70,280), and the ASMR was 1.08 (95% UI: 0.38, 1.79) per 100,000 (Table 1). The number of DALY was 2,730,000 (95% UI: 980,000, 4,525,000), and ASDR was 69.98 (95% UI: 25.05, 116.10) per 100,000 (Supplementary Table S1).

**Figure 2:**
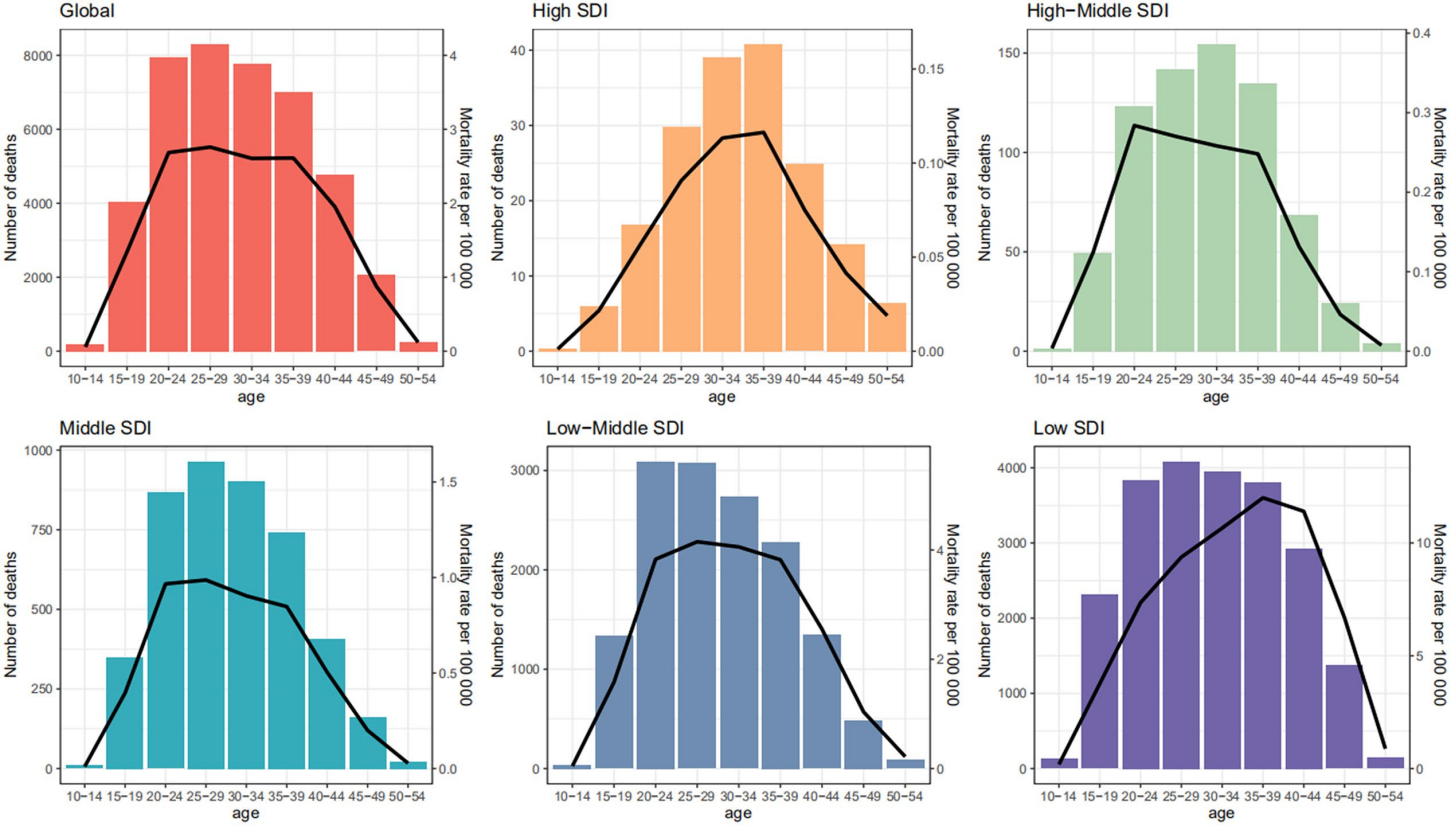
Age-specific numbers (bar plot) and rates (line plot) of deaths of maternal disorders attributable to maternal malnutrition in 2019, by SDI region. SDI, socio-demographic index.

The global age-specific deaths showed a unimodal trend with age, with peak in high SDI region occurring at ages 35–39 years, while those in low-middle and low SDI regions occurred at ages 20–24 and 25–29 years, respectively. Furthermore, the proportion of deaths at ages 15–19 and 20–24 years tended to increase with decreasing SDI levels (Figure 2). DALYs shared the similar patterns, with peaks in high SDI region occurring at ages 30–34 years, and both low-middle and low SDI regions occurring at ages 20–24 years (Supplementary Figure S2).

**Figure 3:**
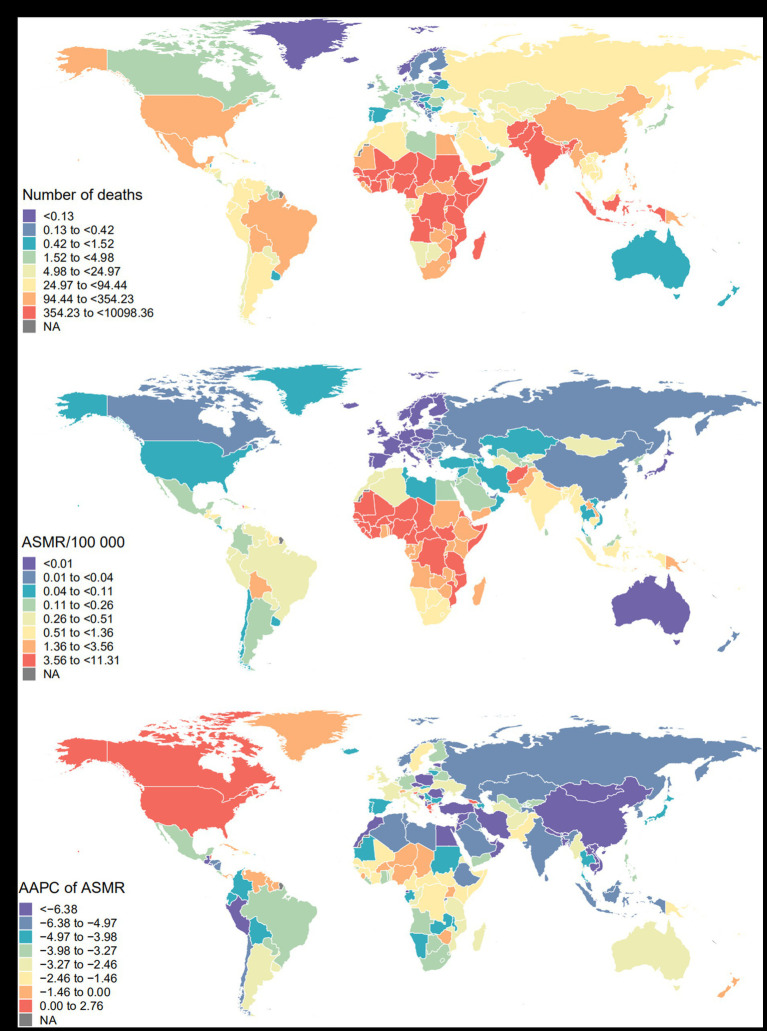
Global mortality burden of maternal disorders attributable to maternal malnutrition. **(A)** Number of deaths in 2019; **(B)** ASMR in 2019; **(C)** AAPC of ASMR from 1990 to 2019. ASMR, age-standardized mortality rate; AAPC, average annual percentage change.

At the SDI region level, low SDI region had the highest number of death, DALY, ASMR, and ASDR at 22,550 (95% UI: 8,060, 37,910), 1,419,000 (95% UI: 504,000, 2,386,000), 4.33 (95% UI: 1.55, 7.28) per 100,000, and 264.07 (95% UI: 94.07, 443.68) per 100,000, respectively (Table 1 and Supplementary Table S1). Among the 21 GBD regions, South Asia had the highest number of death and DALY at 15,160 (95% UI: 5,390, 25,500), 1,003,000 (95% UI: 359,000, 1,669,000), respectively, and ASMR in Central Sub-Saharan Africa (5.11 (95% UI: 1.80, 8.81) per 100,000) highest, ASDR (307.81 (95% UI: 105.73, 521.88)) highest in West Sub-Saharan Africa (Table 1 and Supplementary Table S1). At the country or territory level, the three countries with the highest number of death and DALY were India, Nigeria, and Pakistan, and the three countries with the highest ASMR and ASDR were Chad, Mali, and Somalia (Figures 3A,B and Supplementary Figures S3A,B).

**Figure 4:**
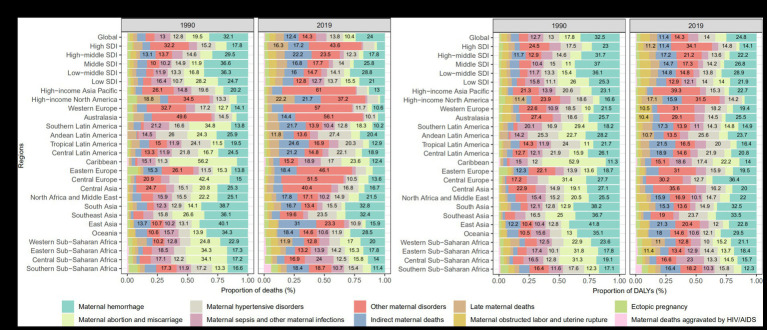
Proportion of deaths **(A)** and DALYs **(B)** of maternal disorders attributable to maternal malnutrition in 1990 and 2019. The labels show the percentage of each disease (greater than 10.0%). DALYs, disability-adjusted life years.

Among the 10 maternal disorders attributable to malnutrition, the three diseases with the highest number of death and DALY were maternal hemorrhage, other maternal disorders, and maternal hypertensive disorders. Maternal hemorrhage accounted for 32.8 and 32.4% of deaths in South and Southeast Asia, respectively; later maternal deaths were high in high-income North America (22.2%); maternal hypertensive disorders accounted for 27.4% of deaths in the Andean region of Latin America; and MAM accounted for 23.6% of deaths in the Caribbean. Similar patterns were observed in DALYs (Figure 4).

### Changing patterns of the global burden of maternal disorders attributable to malnutrition, 1990–2019

The number of global deaths for maternal disorders attributable to malnutrition declined from 73,460 (95% UI: 27,230, 117,530) in 1990 to 42,350 (95% UI: 15,000, 70,280) in 2019, a decrease of 42.35%; ASMR declined form 2.69 (95% UI: 1.00, 4.31) per 100,000 to 1.08 (95% UI: 0.38, 1.79) per 100,000, a decrease of 59.85%, with an AAPC of-3.09 (95% CI: −3.31, −2.88) (Table 1). The number of DALYs declined from 4,672,000 (95% UI: 1,714,000, 7,471,000) in 1990 to 2,728,000 (95% UI: 977,000, 4,525,000) in 2019, a decline of 41.61%; ASDR declined from 168.37 (95% UI: 61.69, 269.52) per 100,000 to 69.98 (95% UI: 25.05, 116.10) per 100,000, a decline of 58.44%, with an AAPC of-2.98 (95% CI: −3.20, −2.77) (Table 1 and Supplementary Table S1).

At the SDI region level, ASMR and ASDR declined in all SDI regions, with the fastest decline in high-middle SDI region, with AAPCs of-5.43 (95% CI: −5.76, −5.10) and-4.84 (95% CI: −5.12, −4.55), respectively. Among the 21 GBD regions, only the high-income North American showed an increase in ASMR and ASDR, while the rest of the regions showed a decrease, with the fastest decline in East Asia, with AAPCs of –8.91 (95% CI: −10.13, −7.67) and –8.16 (95% CI: −9.36, −6.94), respectively (Table 1 and Supplementary Table S1). At the country or territory level, Estonia, Maldives, and China had the fastest ASMR declines, while Maldives, Syrian Arab Republic, and China had the fastest ASDR declines (Figure 3C and Supplementary Figure S3C).

Among the 10 maternal disorders attributable to malnutrition, maternal hemorrhage, MAM, and maternal sepsis and other maternal infections decreased significantly, accounting for 93.50% of the total decline in deaths from 1990 to 2019 (Table 1 and Supplementary Figure S4A); the same pattern was observed in DALYs (Supplementary Table S1 and Supplementary Figure S4B). ASMR and ASDR of the other diseases decreased except for maternal deaths aggravated by HIV/AIDS, and the most rapid decrease was MAM, AAPC was-5.28 (95% CI: −5.40, −5.15) and-5.22 (95% CI: −5.34, −5.09), respectively. (Table 1, Supplementary Table S1, and Supplementary Figures S4C,D).

### The changing patterns at different SDI levels and baseline burden

**Figure 5:**
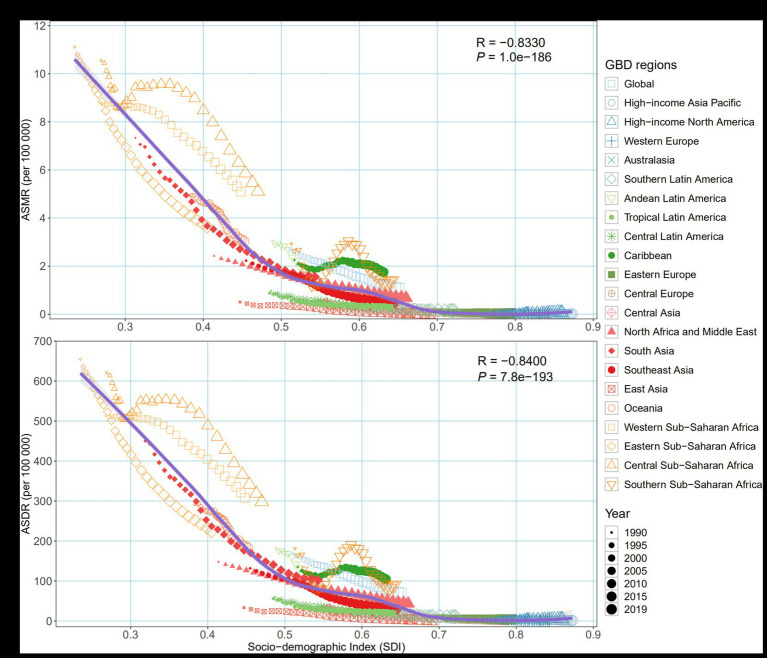
Age-standardized burden rate of maternal disorders attributable to maternal malnutrition across 21 GBD regions by socio-demographic index, 1990–2019. **(A)** ASMR; **(B)** ASDR. The purple line was an adaptive association fitted with adaptive Loess regression based on all data points. GBD, Global Burden of Disease Study; ASMR, age-standardized mortality rate; ASDR, age-standardized disability-adjusted life year rate.

Both ASMR and ASDR showed a significant downward trend with increasing SDI (correlation coefficients around-0.83 and-0.84) (Figure 5). AAPC of ASMR or ASDR showed moderate negative correlation with SDI in 2019 (correlation coefficients around-0.42 and-0.32) (Supplementary Figure S5).

### Predictions of maternal deaths and ASMR attributable to maternal malnutrition

**Figure 6:**
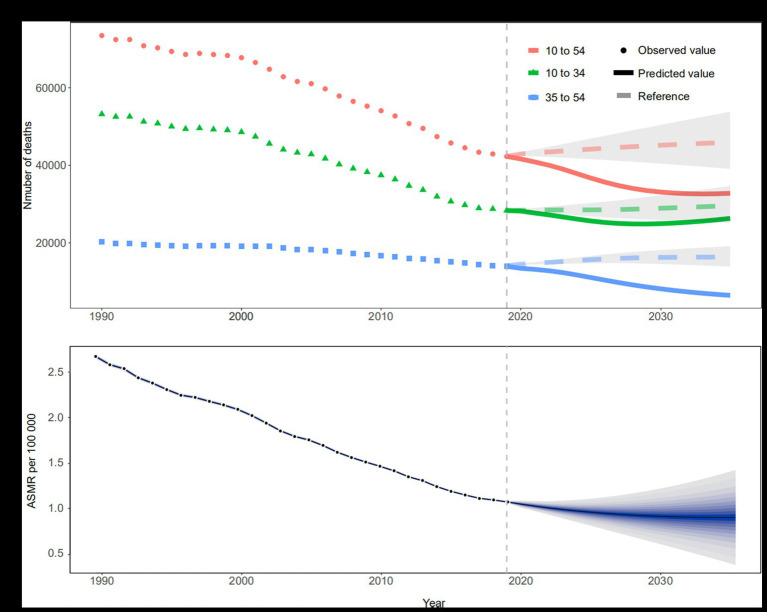
Trends in the number of deaths and ASMR from 1990 to 2035 of maternal disorders attributable to maternal malnutrition. **(A)** Trends in observed (dotted line) and predicted (solid line) number of deaths, shading indicates if the rate remained stable (baseline reference), decreased by 1% per year (optimistic reference, lower limit), and increased by 1% per year (pessimistic reference, upper limit) based on the observed rate in 2019, by age group; **(B)** Trends in observed (black dots) and predicted (solid lines) ASMR, shading from inside to outside represents 5–95% confidence intervals. ASMR, age-standardized mortality rate.

The number of death for the all-age group (10–54) is generally decreasing from 2020–2035, slowing down after 2030, with an overall trend below the positive reference (1% decreased rate annually). The number of death in the 10–34 age group will decrease first and then increase, and the turning point is around 2030, from a near positive reference to a stable reference (mortality rates remain the same as in 2019). For the 35–54 age group, the number of death continues to decline, much lower than the positive reference (Figure 6A). ASMR will continue to decline globally, from 1.08 (95% UI: 0.38, 1.79) in 2019 to 0.88 (95% UI: 0.47, 1.31) in 2035, with all SDI regions decreasing (Figure 6B, Supplementary Table S2, and Supplementary Figure S6).

## Discussion

This study systematically and quantitatively assessed and predicted the global burden of 10 maternal disorders attributable to malnutrition and their spatio-temporal trends. From 1990 to 2019, the number of deaths, DALYs, ASMR and ASDR of maternal disorders attributable to malnutrition showed a downward trend, indicating a reduction in the global burden of related diseases. And the number of deaths and ASMR are predicted to continue to decline from 2020 to 2035. However, the burden of disease remained high in the low SDI regions, especially among young pregnant women aged 20–29 years, and maternal malnutrition should not be underestimated in the context of the global food crisis.

The burden of maternal disease attributable to malnutrition across age groups varies among SDI regions. The study found that the burden of disease was higher among younger pregnant women (20–29 years) in low-and low-middle SDI regions, whereas it was higher among older pregnant women (30–39 years) in high SDI regions. Females in low-and low-middle SDI regions tend to marry and have children at an early age, such as 44 and 39% of girls in South Asia and sub-Saharan Africa, respectively, who marry before the age of 18 years, and are chronically malnourished (22). Evidence has shown that chronic malnutrition delays physical maturation and prolongs the adolescent growth beyond the age of 20 years (23). Therefore, while the nutrition they need for their own growth and development is not guaranteed, they also need additional nutrition to meet the needs of their fetus, which may ultimately exacerbate their own malnutrition. In high SDI countries, older pregnant women (over 35 years) are more likely to be undernourished because women in these countries marry and give birth later, such as in Germany and the United Kingdom, where the average age at birth is 31.50 and 30.48 years, respectively (24, 25). At the same time, there is also a preference for foods high in fat, sugar, and salt (26, 27). As people grow older, they may face more chronic diseases or metabolic diseases, such as hypertension and diabetes, which gradually reduce the body’s metabolism and absorption capacity, leading to malnutrition (28).

The burden of maternal disease attributable to malnutrition is negatively associated with SDI, which is consistent with the WHO study (29). The burden of disease is higher in sub-Saharan Africa and South Asia, where a combination of factors, such as backward economic development, suboptimal diets, and extreme climatic environments, prevent pregnant women from obtaining adequate, high-quality nutrients (30, 31). For example, the latest data from UNICEF show that 10 and 23% of women aged >18 years in sub-Saharan Africa and Southern Asia, respectively, have a low BMI (18.5 kg/m^2^), much higher than in Western Europe (2%) and North America (2%) (32). This study found that the burden of disease in East Asia decreased fastest. This was made possible by a series of policies implemented by the UNICEF East Asia and Pacific Regional Office to prevent malnutrition among women, such as the production of “Food and Nutrition Security Country Profiles” for 29 countries in East Asia and the Pacific in collaboration with FAO, and the promotion of legislation related to nutrition security for pregnant women (33). As the largest country in East Asia, China, with the development of economy and the improvement of people’s living standards, the government has also taken a series of nutrition improvement measures, including promoting scientific diet, strengthening food safety supervision, and carrying out nutrition education, which has significantly improved the nutritional intake and dietary structure of pregnant women. It has made a significant contribution to the rapid decline in the incidence of malnutrition (34). From 2002 to 2020, the incidence of anemia in pregnant women in China decreased from 22.5 to 13.6% (35, 36). Work to reduce inequalities was also ongoing worldwide, with the UN’s proposed Decade of Action on Nutrition in 2016 providing an enabling environment for all countries, regardless of their income levels and the characteristics of their food and health systems, to ensure that governments and stakeholders take action to develop and implement programs aimed at eliminating undernutrition (37).

The burden of maternal hemorrhage accounted for the highest proportion of the 10 maternal diseases attributable to malnutrition. WHO studies have shown that at least 30–40% of pregnant women are iron deficient (38), and almost all women studied (97.5%) had a total dietary folic acid intake that was lower than the WHO-recommended intake of folic acid during pregnancy and lactation (39). When pregnant women have insufficient dietary iron and folic acid intake, nutritional anemia often occurs, causing changes such as compensatory placental hypertrophy, leading to uterine inertia, and ultimately increasing the risk of postpartum hemorrhage (40). Higher proportion of late maternal deaths were found in high-income North America. The National Institute of Perinatal Epidemiology at the University of Oxford categorized the causes of late maternal deaths into four main groups: cardiovascular disease, thromboembolism, cancer, and suicide (41), and over-nutrition is more prevalent in high-income North America, with approximately 86 million adults in the United States aged 20 years and older having a total cholesterol level of more than 200 mg/dL (42), putting increased risk of cardiovascular diseases such as atherosclerosis and perinatal cardiomyopathy, which usually appear after only 1 month postpartum, thus contributing to late maternal mortality (43). The higher burden of maternal hypertension attributable to malnutrition in the Andean Latin American may be related to the increase of high-sugar, high-salt, and high-fat diets and ultra-processed foods in the region (44), from 2000 to 2013, the retail sales of ultra-processed products in Bolivia, Ecuador and Peru all increased, and Peru even increased by four times (45). It has been suggested that a low-sugar, low-fat, low-protein, and low-sodium dietary pattern may reduce the risk of hypertensive disorders during pregnancy by improving endothelial cell function, reducing oxidative stress, and having a positive effect on the renin-angiotensin-aldosterone system, thereby reducing the risk of hypertensive disorders in pregnancy (46). The high proportion of the burden of MAM in the Caribbean may be due to the predominance of refined grains in the staple diet of the region’s inhabitants and the low intake of fresh vegetables and fruits (such as Haiti, Jamaica, Cuba, Puerto Rico and the Dominican Republic) (47–49), resulting in insufficient dietary folate intake and an increase in plasma total homocysteine concentration, which may cause embryotoxicity and increase the risk of abortion by interfering with the body’s methylation reaction, damaging DNA, and destroying the normal cycle of embryonic cells (50, 51). In conclusion, malnutrition affects pregnant women differently at different ages and in different SDI regions. Targeted nutritional guidance and disease prevention strategies should be taken according to the specificity of their age, region and disease, such as the Food Systems Summit convened by the United Nations to seek solutions and action for all to change the way the world produces, consumes and thinks about food, thereby safeguarding health and advancing the SDGs (52).

There are some limitations in our study. First, all the data used in our study were obtained from GBD2019, which collects data from different sources, and therefore, there may be inconsistencies and incompatibilities between these data, which may result in bias. Second, our predictions were based on the BAPC model, which only simulates changes in age, period, and cohort effects in the current context, it may lead to less precise predictions. In addition, a comprehensive evaluation of the burden of disease should also include an evaluation of economic, household, and social dimensions, so multidimensional analyses should be added in future studies to provide more accurate results.

## Conclusion

In summary, this study found that maternal malnutrition health is improving globally, but the burden of maternal disorders attributable to malnutrition remains high in low SDI regions and young pregnant women. Therefore, in countries and territories with low SDI, more attention should be paid to the development of preventive measures for maternal malnutrition, such as guaranteeing the food supply, carrying out nutritional education, and strengthening food safety regulation, etc. Only through an approach involving multi-sectoral cooperation across health, education, social protection, agriculture and other sectors to continuously reduce the burden of maternal disorders attributable to malnutrition, can the goal of eliminating all forms of hunger and malnutrition proposed in the UN SDGs be achieved as soon as possible.

## Data availability statement

The original contributions presented in the study are included in the article/Supplementary material, further inquiries can be directed to the corresponding authors.

## Author contributions

TX: Data curation, Formal analysis, Investigation, Software, Visualization, Writing – original draft, Writing – review & editing. CD: Data curation, Formal analysis, Investigation, Software, Visualization, Writing – original draft, Writing – review & editing. JS: Data curation, Formal analysis, Investigation, Software, Visualization, Writing – original draft, Writing – review & editing. CH: Visualization, Writing – review & editing. ZC: Data curation, Formal analysis, Methodology, Visualization, Writing – original draft. ZS: Conceptualization, Data curation, Formal analysis, Methodology, Visualization, Writing – original draft. TY: Data curation, Methodology, Visualization, Writing – original draft. CG: Data curation, Formal analysis, Methodology, Visualization, Writing – original draft. WW: Data curation, Formal analysis, Methodology, Visualization, Writing – original draft. DR: Data curation, Formal analysis, Methodology, Visualization, Writing – original draft. XL: Data curation, Formal analysis, Methodology, Visualization, Writing – original draft. YH: Data curation, Formal analysis, Methodology, Visualization, Writing – original draft. JM: Conceptualization, Data curation, Funding acquisition, Methodology, Project administration, Visualization, Writing – review & editing. QN: Conceptualization, Data curation, Funding acquisition, Methodology, Project administration, Visualization, Writing – review & editing. YY: Conceptualization, Data curation, Funding acquisition, Methodology, Project administration, Visualization, Writing – review & editing.

## Footnotes

^1^http://ghdx.healthdata.org/gbd-results-tool

^2^https://ghdx.healthdata.org/record/ihme-data/global-population-forecasts-2017-2100

^3^https://www.r-project.org/
