# Supplementary material for: Global burden of maternal disorders attributable to malnutrition from 1990 to 2019 and predictions to 2035: worsening or improving?
Source: Front Nutr. 2024 Feb 15;11:1343772. doi: 10.3389/fnut.2024.1343772 (PMC10902107; doi:10.3389/fnut.2024.1343772)
Supplement: Supplementary file 1 [file Data_Sheet_1.docx]

**Table S1.** Global DALYs of maternal disorders attributable to child and maternal malnutrition in 1990 and 2019, and the temporal trend from 1990 to 2019.

| **Characteristics** | **1990** | | |  | **2019** | | |  | **1990-2019** | |
| --- | --- | --- | --- | --- | --- | --- | --- | --- | --- | --- |
|  | **DALYs**  **No.×10^5^**  **（95%UI）** | **ASDR per**  **100 000**  **(95%UI)** | **Age-**  **standardized**  **PAF, %**  **(95%UI)** |  | **DALYs**  **No.×10^5^**  **（95%UI）** | **ASDR per**  **100 000**  **(95%UI)** | **Age-**  **standardized PAF, %**  **(95%UI)** |  | **AAPC of**  **ASDR**  **(95%CI)** | **AAPC of Age-**  **standardized**  **PAF(95%CI)** |
| **Overall** | 46.72 (17.14 , 74.71) | 168.37 (61.69 , 269.52) | 24.22 (8.97 , 38.44) |  | 27.28 (9.77 , 45.25) | 69.98 (25.05 , 116.10) | 21.52 (7.93 , 34.45) |  | -2.98 (-3.20 , -2.77) | -0.41 (-0.45 , -0.36) |
| **Disease type** |  |  |  |  |  |  |  |  |  |  |
| Maternal hemorrhage | 15.17 (5.61 , 24.65) | 54.45 (20.11 , 88.50) | 24.70 (9.16 , 39.35) |  | 6.77 (2.42 , 11.34) | 17.36 (6.20 , 29.08) | 21.90 (8.05 , 35.04) |  | -3.89 (-4.19 , -3.58) | -0.42 (-0.47 , -0.36) |
| Maternal sepsis and other maternal infections | 5.93 (2.26 , 9.60) | 21.16 (8.00 , 34.38) | 24.92 (9.26 , 39.50) |  | 2.34 (0.86 , 3.91) | 6.02 (2.20 , 10.07) | 22.02 (8.10 , 35.31) |  | -4.23 (-4.37 , -4.10) | -0.42 (-0.45 , -0.40) |
| Maternal hypertensive disorders | 6.06 (2.22 , 9.73) | 21.42 (7.85 , 34.33) | 23.48 (8.64 , 37.44) |  | 3.82 (1.36 , 6.39) | 9.84 (3.51 , 16.50) | 20.92 (7.69 , 33.65) |  | -2.63 (-2.75 , -2.50) | -0.40 (-0.45 , -0.34) |
| Maternal obstructed labor and uterine rupture | 2.28 (0.81 , 3.82) | 8.46 (3.00 , 14.20) | 24.42 (9.02 , 38.68) |  | 2.15 (0.76 , 3.59) | 5.45 (1.93 , 9.14) | 21.47 (7.90 , 34.48) |  | -1.52 (-1.68 , -1.37) | -0.44 (-0.46 , -0.42) |
| Maternal abortion and miscarriage | 8.30 (3.06 , 13.50) | 30.68 (11.32 , 50.04) | 24.00 (8.80 , 38.18) |  | 2.52 (0.89 , 4.21) | 6.45 (2.28 , 10.78) | 22.33 (8.23 , 35.66) |  | -5.22 (-5.34 , -5.09) | -0.24 (-0.27 , -0.22) |
| Ectopic pregnancy | 0.81 (0.30 , 1.32) | 2.97 (1.09 , 4.83) | 23.87 (8.76 , 38.07) |  | 0.83 (0.30 , 1.39) | 2.12 (0.76 , 3.55) | 21.91 (8.01 , 35.13) |  | -1.16 (-1.42 , -0.90) | -0.30 (-0.33 , -0.26) |
| Indirect maternal deaths | 3.12 (1.14 , 5.10) | 11.03 (4.01 , 18.06) | 24.04 (8.93 , 38.32) |  | 3.11 (1.09 , 5.16) | 8.02 (2.81 , 13.27) | 20.89 (7.68 , 33.28) |  | -1.04 (-1.40 , -0.67) | -0.48 (-0.54 , -0.43) |
| Late maternal deaths | 1.39 (0.51 , 2.28) | 5.05 (1.84 , 8.30) | 23.90 (8.93 , 37.94) |  | 1.65 (0.57 , 2.84) | 4.24 (1.46 , 7.27) | 21.56 (7.94 , 34.42) |  | -0.60 (-0.72 , -0.48) | -0.36 (-0.38 , -0.33) |
| Maternal deaths aggravated by HIV/AIDS | 0.11 (0.04 , 0.19) | 0.39 (0.14 , 0.68) | 24.98 (9.34 , 39.52) |  | 0.19 (0.07 , 0.35) | 0.48 (0.17 , 0.87) | 22.13 (8.11 , 35.52) |  | 0.74 (0.59 , 0.90) | -0.42 (-0.43 , -0.40) |
| Other maternal disorders | 3.54 (1.32 , 5.72) | 12.76 (4.80 , 20.54) | 23.10 (8.51 , 36.75) |  | 3.90 (1.36 , 6.49) | 10.00 (3.49 , 16.64) | 21.10 (7.71 , 33.75) |  | -0.85 (-1.07 , -0.63) | -0.31 (-0.38 , -0.24) |
| **SDI region** |  |  |  |  |  |  |  |  |  |  |
| High SDI | 0.19 (0.07 , 0.32) | 4.47 (1.56 , 7.46) | 12.21 (4.35 , 20.01) |  | 0.14 (0.05 , 0.24) | 3.10 (1.10 , 5.26) | 10.97 (3.97 , 17.98) |  | -1.25 (-1.66 , -0.83) | -0.37 (-0.40 , -0.33) |
| High-middle SDI | 2.02 (0.73 , 3.42) | 32.46 (11.69 , 54.87) | 19.25 (6.93 , 31.38) |  | 0.54 (0.19 , 0.88) | 7.82 (2.78 , 12.81) | 15.57 (5.62 , 25.19) |  | -4.84 (-5.12 , -4.55) | -0.74 (-0.89 , -0.58) |
| Middle SDI | 8.36 (3.03 , 13.80) | 90.37 (32.81 , 149.00) | 19.91 (7.21 , 32.24) |  | 3.01 (1.10 , 4.93) | 24.21 (8.84 , 39.61) | 18.11 (6.57 , 29.28) |  | -4.42 (-4.95 , -3.89) | -0.33 (-0.41 , -0.25) |
| Low-middle SDI | 20.78 (7.53 , 33.24) | 373.42 (135.21 , 597.79) | 25.48 (9.55 , 40.42) |  | 9.38 (3.35 , 15.44) | 98.79 (35.32 , 162.88) | 21.26 (7.76 , 33.98) |  | -4.48 (-4.84 , -4.11) | -0.63 (-0.69 , -0.57) |
| Low SDI | 15.34 (5.75 , 24.88) | 659.38 (247.36 , 1069.16) | 26.62 (9.96 , 41.96) |  | 14.19 (5.04 , 23.86) | 264.07 (94.07 , 443.68) | 22.98 (8.47 , 36.78) |  | -3.11 (-3.21 , -3.00) | -0.50 (-0.53 , -0.48) |
| **GBD Region** |  |  |  |  |  |  |  |  |  |  |
| High-income Asia Pacific | 0.04 (0.01 , 0.06) | 4.37 (1.55 , 7.27) | 15.13 (5.41 , 24.87) |  | 0.01 (0.00 , 0.02) | 1.39 (0.50 , 2.41) | 12.29 (4.40 , 20.30) |  | -3.95 (-4.69 , -3.20) | -0.72 (-0.79 , -0.65) |
| High-income North America | 0.06 (0.02 , 0.10) | 3.94 (1.37 , 6.61) | 10.95 (3.90 , 17.91) |  | 0.08 (0.03 , 0.14) | 4.99 (1.75 , 8.34) | 11.24 (4.06 , 18.72) |  | 0.79 (-0.12 , 1.72) | 0.09 (0.07 , 0.11) |
| Western Europe | 0.04 (0.02 , 0.08) | 2.30 (0.82 , 3.92) | 9.66 (3.45 , 15.68) |  | 0.03 (0.01 , 0.04) | 1.37 (0.49 , 2.36) | 8.81 (3.20 , 14.35) |  | -1.80 (-2.26 , -1.34) | -0.32 (-0.37 , -0.26) |
| Australasia | 0.00 (0.00 , 0.01) | 3.36 (1.19 , 5.85) | 11.45 (4.18 , 18.90) |  | 0.00 (0.00 , 0.01) | 2.29 (0.78 , 4.06) | 10.62 (3.87 , 17.52) |  | -1.09 (-1.40 , -0.77) | -0.26 (-0.28 , -0.25) |
| Southern Latin America | 0.06 (0.02 , 0.09) | 22.46 (8.08 , 36.33) | 13.27 (4.79 , 21.53) |  | 0.04 (0.01 , 0.06) | 10.84 (3.92 , 18.15) | 11.62 (4.29 , 19.08) |  | -2.60 (-2.96 , -2.23) | -0.46 (-0.49 , -0.42) |
| Andean Latin America | 0.34 (0.12 , 0.57) | 181.81 (64.51 , 303.14) | 21.17 (7.61 , 34.43) |  | 0.13 (0.04 , 0.22) | 37.89 (12.85 , 65.66) | 15.03 (5.42 , 24.66) |  | -5.28 (-5.85 , -4.72) | -1.19 (-1.24 , -1.13) |
| Tropical Latin America | 0.50 (0.18 , 0.84) | 60.60 (21.64 , 101.75) | 19.71 (6.93 , 33.25) |  | 0.23 (0.08 , 0.39) | 19.61 (6.90 , 32.70) | 16.89 (6.06 , 28.04) |  | -3.57 (-4.06 , -3.08) | -0.53 (-0.55 , -0.51) |
| Central Latin America | 0.49 (0.17 , 0.80) | 55.74 (19.87 , 91.57) | 15.87 (5.71 , 26.11) |  | 0.26 (0.09 , 0.44) | 19.03 (6.50 , 32.19) | 13.12 (4.68 , 21.51) |  | -3.49 (-3.99 , -2.99) | -0.65 (-0.71 , -0.58) |
| Caribbean | 0.25 (0.09 , 0.42) | 134.76 (50.20 , 226.26) | 23.95 (8.88 , 38.55) |  | 0.25 (0.08 , 0.44) | 104.42 (34.58 , 179.73) | 21.12 (7.69 , 34.36) |  | -0.87 (-1.16 , -0.58) | -0.43 (-0.46 , -0.40) |
| Eastern Europe | 0.13 (0.04 , 0.22) | 11.59 (3.99 , 19.46) | 13.00 (4.62 , 21.23) |  | 0.04 (0.01 , 0.07) | 4.19 (1.45 , 7.36) | 11.72 (4.19 , 19.45) |  | -3.46 (-4.86 , -2.03) | -0.36 (-0.40 , -0.31) |
| Central Europe | 0.06 (0.02 , 0.09) | 9.36 (3.25 , 15.60) | 14.27 (5.02 , 23.20) |  | 0.01 (0.00 , 0.02) | 2.67 (0.95 , 4.67) | 12.42 (4.42 , 20.36) |  | -4.21 (-4.91 , -3.51) | -0.47 (-0.52 , -0.42) |
| Central Asia | 0.14 (0.05 , 0.22) | 37.56 (13.25 , 60.93) | 19.97 (7.15 , 32.42) |  | 0.07 (0.03 , 0.12) | 13.92 (4.96 , 23.50) | 17.67 (6.48 , 28.69) |  | -3.39 (-4.19 , -2.59) | -0.43 (-0.46 , -0.39) |
| North Africa and Middle East | 2.30 (0.83 , 3.77) | 147.74 (53.71 , 242.46) | 20.64 (7.57 , 32.94) |  | 1.41 (0.50 , 2.41) | 43.43 (15.41 , 74.49) | 18.97 (6.97 , 30.62) |  | -4.12 (-4.24 , -4.00) | -0.29 (-0.32 , -0.25) |
| South Asia | 25.08 (9.17 , 40.35) | 466.29 (170.90 , 751.67) | 27.36 (10.31 , 43.28) |  | 10.03 (3.59 , 16.69) | 102.10 (36.40 , 169.98) | 22.83 (8.37 , 36.52) |  | -5.11 (-5.44 , -4.77) | -0.62 (-0.66 , -0.59) |
| Southeast Asia | 3.20 (1.18 , 5.31) | 131.53 (48.47 , 218.73) | 18.42 (6.58 , 29.59) |  | 1.04 (0.37 , 1.74) | 28.79 (10.37 , 48.44) | 15.42 (5.60 , 25.19) |  | -5.11 (-5.26 , -4.97) | -0.61 (-0.64 , -0.58) |
| East Asia | 2.38 (0.82 , 4.16) | 33.46 (11.70 , 58.58) | 18.99 (6.87 , 31.89) |  | 0.20 (0.07 , 0.35) | 2.81 (0.97 , 4.94) | 13.40 (4.79 , 22.08) |  | -8.16 (-9.36 , -6.94) | -1.19 (-1.23 , -1.16) |
| Oceania | 0.09 (0.03 , 0.16) | 290.81 (109.01 , 495.37) | 24.78 (8.95 , 40.19) |  | 0.12 (0.04 , 0.22) | 181.91 (64.36 , 318.01) | 22.34 (8.00 , 36.35) |  | -1.63 (-1.82 , -1.44) | -0.36 (-0.37 , -0.34) |
| Western Sub-Saharan Africa | 4.28 (1.56 , 7.00) | 512.96 (188.85 , 844.81) | 23.14 (8.56 , 36.92) |  | 6.76 (2.33 , 11.43) | 307.81 (105.73 , 521.88) | 23.62 (8.77 , 37.47) |  | -1.73 (-1.80 , -1.66) | 0.07 (0.05 , 0.10) |
| Eastern Sub-Saharan Africa | 5.32 (1.98 , 8.78) | 654.09 (241.42 , 1080.68) | 24.55 (9.01 , 39.00) |  | 4.38 (1.57 , 7.36) | 223.79 (80.07 , 378.44) | 21.06 (7.70 , 33.88) |  | -3.63 (-3.75 , -3.51) | -0.53 (-0.54 , -0.51) |
| Central Sub-Saharan Africa | 1.48 (0.55 , 2.51) | 620.85 (231.29 , 1057.72) | 28.06 (10.89 , 44.09) |  | 1.79 (0.63 , 3.03) | 297.94 (105.43 , 507.15) | 21.82 (7.98 , 35.32) |  | -2.55 (-2.80 , -2.30) | -0.87 (-0.91 , -0.83) |
| Southern Sub-Saharan Africa | 0.49 (0.18 , 0.81) | 180.86 (64.89 , 297.03) | 21.43 (7.69 , 35.10) |  | 0.40 (0.14 , 0.67) | 90.16 (32.91 , 152.17) | 20.58 (7.45 , 33.60) |  | -2.33 (-3.68 , -0.96) | -0.14 (-0.20 , -0.08) |

Note: DALYs, disability-adjusted life years; ASDR, age-standardized disability-adjusted life year rate; PAF, population attributable fraction; AAPC, average annual percentage change; UI, uncertainty interval; CI, confidence interval; SDI, socio-demographic index; GBD, Global Burden of Disease Study.

**Table S2.** The number of maternal deaths and age-standardized mortality rates attributable to maternal malnutrition predicted from 2020-2035 at the global level and in the different SDI regions.

| **Region** | **Number of deaths** | | | |  | **ASMR per 100 000** | | | |
| --- | --- | --- | --- | --- | --- | --- | --- | --- | --- |
|  | **2020** | **2025** | **2030** | **2035** |  | **2020** | **2025** | **2030** | **2035** |
| Global | 42943.08 | 36940.41 | 33148.31 | 31462.14 |  | 1.05 | 0.95 | 0.90 | 0.88 |
| High SDI | 161.91 | 63.89 | <0.01 | <0.01 |  | 0.04 | 0.03 | 0.03 | 0.02 |
| High-middle SDI | 323.82 | 68.09 | <0.01 | <0.01 |  | 0.10 | 0.08 | 0.07 | 0.06 |
| Middle SDI | 4968.86 | 3303.35 | 2677.26 | 2778.56 |  | 0.34 | 0.32 | 0.30 | 0.27 |
| Low-middle SDI | 18116.31 | 15245.39 | 13017.86 | 11638.32 |  | 1.47 | 1.23 | 1.04 | 0.89 |
| Low SDI | 19372.18 | 18259.69 | 17453.19 | 17045.26 |  | 4.12 | 3.36 | 2.80 | 2.38 |

Note: SDI, socio-demographic index; ASMR, age-standardized mortality rate.

| **Table S3. Classification of 21 GBD regions and 7 Super GBD regions, and SDI values for 204 countries.** | | | | |
| --- | --- | --- | --- | --- |
| Location | Regions | Super GBD regions | SDI | SDI regions |
| Afghanistan | North Africa and Middle East | North Africa and Middle East | 0.343 | Low SDI |
| Albania | Central Europe | Central Europe, eastern Europe, and central Asia | 0.681 | Middle SDI |
| Algeria | North Africa and Middle East | North Africa and Middle East | 0.652 | Middle SDI |
| American Samoa | Oceania | Southeast Asia, east Asia, and Oceania | 0.712 | High-middle SDI |
| Andorra | Western Europe | High income | 0.894 | High SDI |
| Angola | Central Sub-Saharan Africa | Sub-Saharan Africa | 0.470 | Low-middle SDI |
| Antigua and Barbuda | Caribbean | Latin America and Caribbean | 0.743 | High-middle SDI |
| Argentina | Southern Latin America | Latin America and Caribbean | 0.708 | High-middle SDI |
| Armenia | Central Asia | Central Europe, eastern Europe, and central Asia | 0.689 | Middle SDI |
| Australia | Australasia | High income | 0.839 | High SDI |
| Austria | Western Europe | High income | 0.849 | High SDI |
| Azerbaijan | Central Asia | Central Europe, eastern Europe, and central Asia | 0.683 | Middle SDI |
| Bahamas | Caribbean | Latin America and Caribbean | 0.796 | High-middle SDI |
| Bahrain | North Africa and Middle East | North Africa and Middle East | 0.751 | High-middle SDI |
| Bangladesh | South Asia | South Asia | 0.483 | Low-middle SDI |
| Barbados | Caribbean | Latin America and Caribbean | 0.742 | High-middle SDI |
| Belarus | Eastern Europe | Central Europe, eastern Europe, and central Asia | 0.745 | High-middle SDI |
| Belgium | Western Europe | High income | 0.851 | High SDI |
| Belize | Caribbean | Latin America and Caribbean | 0.603 | Low-middle SDI |
| Benin | Western Sub-Saharan Africa | Sub-Saharan Africa | 0.352 | Low SDI |
| Bermuda | Caribbean | Latin America and Caribbean | 0.813 | High SDI |
| Bhutan | South Asia | South Asia | 0.455 | Low-middle SDI |
| Bolivia (Plurinational State of) | Andean Latin America | Latin America and Caribbean | 0.566 | Low-middle SDI |
| Bosnia and Herzegovina | Central Europe | Central Europe, eastern Europe, and central Asia | 0.718 | High-middle SDI |
| Botswana | Southern Sub-Saharan Africa | Sub-Saharan Africa | 0.634 | Middle SDI |
| Brazil | Tropical Latin America | Latin America and Caribbean | 0.640 | Middle SDI |
| Brunei Darussalam | High-income Asia Pacific | High income | 0.823 | High SDI |
| Bulgaria | Central Europe | Central Europe, eastern Europe, and central Asia | 0.764 | High-middle SDI |
| Burkina Faso | Western Sub-Saharan Africa | Sub-Saharan Africa | 0.257 | Low SDI |
| Burundi | Eastern Sub-Saharan Africa | Sub-Saharan Africa | 0.284 | Low SDI |
| Cabo Verde | Western Sub-Saharan Africa | Sub-Saharan Africa | 0.525 | Low-middle SDI |
| Cambodia | Southeast Asia | Southeast Asia, east Asia, and Oceania | 0.469 | Low-middle SDI |
| Cameroon | Western Sub-Saharan Africa | Sub-Saharan Africa | 0.490 | Low-middle SDI |
| Canada | High-income North America | High income | 0.873 | High SDI |
| Central African Republic | Central Sub-Saharan Africa | Sub-Saharan Africa | 0.274 | Low SDI |
| Chad | Western Sub-Saharan Africa | Sub-Saharan Africa | 0.238 | Low SDI |
| Chile | Southern Latin America | Latin America and Caribbean | 0.759 | High-middle SDI |
| China | East Asia | Southeast Asia, east Asia, and Oceania | 0.686 | Middle SDI |
| Colombia | Central Latin America | Latin America and Caribbean | 0.633 | Middle SDI |
| Comoros | Eastern Sub-Saharan Africa | Sub-Saharan Africa | 0.455 | Low-middle SDI |
| Congo | Central Sub-Saharan Africa | Sub-Saharan Africa | 0.568 | Low-middle SDI |
| Cook Islands | Oceania | Southeast Asia, east Asia, and Oceania | 0.764 | High-middle SDI |
| Costa Rica | Central Latin America | Latin America and Caribbean | 0.680 | Middle SDI |
| Côte d'Ivoire | Western Sub-Saharan Africa | Sub-Saharan Africa | 0.408 | Low SDI |
| Croatia | Central Europe | Central Europe, eastern Europe, and central Asia | 0.794 | High-middle SDI |
| Cuba | Caribbean | Latin America and Caribbean | 0.668 | Middle SDI |
| Cyprus | Western Europe | High income | 0.841 | High SDI |
| Czechia | Central Europe | Central Europe, eastern Europe, and central Asia | 0.828 | High SDI |
| Democratic People's Republic of Korea | East Asia | Southeast Asia, east Asia, and Oceania | 0.558 | Low-middle SDI |
| Democratic Republic of the Congo | Central Sub-Saharan Africa | Sub-Saharan Africa | 0.382 | Low SDI |
| Denmark | Western Europe | High income | 0.890 | High SDI |
| Djibouti | Eastern Sub-Saharan Africa | Sub-Saharan Africa | 0.459 | Low-middle SDI |
| Dominica | Caribbean | Latin America and Caribbean | 0.729 | High-middle SDI |
| Dominican Republic | Caribbean | Latin America and Caribbean | 0.592 | Low-middle SDI |
| Ecuador | Andean Latin America | Latin America and Caribbean | 0.640 | Middle SDI |
| Egypt | North Africa and Middle East | North Africa and Middle East | 0.658 | Middle SDI |
| El Salvador | Central Latin America | Latin America and Caribbean | 0.573 | Low-middle SDI |
| Equatorial Guinea | Central Sub-Saharan Africa | Sub-Saharan Africa | 0.685 | Middle SDI |
| Eritrea | Eastern Sub-Saharan Africa | Sub-Saharan Africa | 0.396 | Low SDI |
| Estonia | Eastern Europe | Central Europe, eastern Europe, and central Asia | 0.835 | High SDI |
| Eswatini | Southern Sub-Saharan Africa | Sub-Saharan Africa | 0.577 | Low-middle SDI |
| Ethiopia | Eastern Sub-Saharan Africa | Sub-Saharan Africa | 0.343 | Low SDI |
| Fiji | Oceania | Southeast Asia, east Asia, and Oceania | 0.664 | Middle SDI |
| Finland | Western Europe | High income | 0.856 | High SDI |
| France | Western Europe | High income | 0.834 | High SDI |
| Gabon | Central Sub-Saharan Africa | Sub-Saharan Africa | 0.656 | Middle SDI |
| Gambia | Western Sub-Saharan Africa | Sub-Saharan Africa | 0.399 | Low SDI |
| Georgia | Central Asia | Central Europe, eastern Europe, and central Asia | 0.702 | High-middle SDI |
| Germany | Western Europe | High income | 0.898 | High SDI |
| Ghana | Western Sub-Saharan Africa | Sub-Saharan Africa | 0.557 | Low-middle SDI |
| Greece | Western Europe | High income | 0.794 | High-middle SDI |
| Greenland | High-income North America | High income | 0.761 | High-middle SDI |
| Grenada | Caribbean | Latin America and Caribbean | 0.669 | Middle SDI |
| Guam | Oceania | Southeast Asia, east Asia, and Oceania | 0.813 | High SDI |
| Guatemala | Central Latin America | Latin America and Caribbean | 0.526 | Low-middle SDI |
| Guinea | Western Sub-Saharan Africa | Sub-Saharan Africa | 0.325 | Low SDI |
| Guinea-Bissau | Western Sub-Saharan Africa | Sub-Saharan Africa | 0.355 | Low SDI |
| Guyana | Caribbean | Latin America and Caribbean | 0.618 | Middle SDI |
| Haiti | Caribbean | Latin America and Caribbean | 0.432 | Low SDI |
| Honduras | Central Latin America | Latin America and Caribbean | 0.496 | Low-middle SDI |
| Hungary | Central Europe | Central Europe, eastern Europe, and central Asia | 0.791 | High-middle SDI |
| Iceland | Western Europe | High income | 0.869 | High SDI |
| India | South Asia | South Asia | 0.566 | Low-middle SDI |
| Indonesia | Southeast Asia | Southeast Asia, east Asia, and Oceania | 0.660 | Middle SDI |
| Iran (Islamic Republic of) | North Africa and Middle East | North Africa and Middle East | 0.670 | Middle SDI |
| Iraq | North Africa and Middle East | North Africa and Middle East | 0.671 | Middle SDI |
| Ireland | Western Europe | High income | 0.867 | High SDI |
| Israel | Western Europe | High income | 0.803 | High-middle SDI |
| Italy | Western Europe | High income | 0.801 | High-middle SDI |
| Jamaica | Caribbean | Latin America and Caribbean | 0.684 | Middle SDI |
| Japan | High-income Asia Pacific | High income | 0.870 | High SDI |
| Jordan | North Africa and Middle East | North Africa and Middle East | 0.731 | High-middle SDI |
| Kazakhstan | Central Asia | Central Europe, eastern Europe, and central Asia | 0.723 | High-middle SDI |
| Kenya | Eastern Sub-Saharan Africa | Sub-Saharan Africa | 0.508 | Low-middle SDI |
| Kiribati | Oceania | Southeast Asia, east Asia, and Oceania | 0.527 | Low-middle SDI |
| Kuwait | North Africa and Middle East | North Africa and Middle East | 0.851 | High SDI |
| Kyrgyzstan | Central Asia | Central Europe, eastern Europe, and central Asia | 0.596 | Low-middle SDI |
| Lao People's Democratic Republic | Southeast Asia | Southeast Asia, east Asia, and Oceania | 0.490 | Low-middle SDI |
| Latvia | Eastern Europe | Central Europe, eastern Europe, and central Asia | 0.820 | High SDI |
| Lebanon | North Africa and Middle East | North Africa and Middle East | 0.708 | High-middle SDI |
| Lesotho | Southern Sub-Saharan Africa | Sub-Saharan Africa | 0.507 | Low-middle SDI |
| Liberia | Western Sub-Saharan Africa | Sub-Saharan Africa | 0.370 | Low SDI |
| Libya | North Africa and Middle East | North Africa and Middle East | 0.709 | High-middle SDI |
| Lithuania | Eastern Europe | Central Europe, eastern Europe, and central Asia | 0.843 | High SDI |
| Luxembourg | Western Europe | High income | 0.895 | High SDI |
| Madagascar | Eastern Sub-Saharan Africa | Sub-Saharan Africa | 0.396 | Low SDI |
| Malawi | Eastern Sub-Saharan Africa | Sub-Saharan Africa | 0.384 | Low SDI |
| Malaysia | Southeast Asia | Southeast Asia, east Asia, and Oceania | 0.737 | High-middle SDI |
| Maldives | Southeast Asia | Southeast Asia, east Asia, and Oceania | 0.562 | Low-middle SDI |
| Mali | Western Sub-Saharan Africa | Sub-Saharan Africa | 0.263 | Low SDI |
| Malta | Western Europe | High income | 0.801 | High-middle SDI |
| Marshall Islands | Oceania | Southeast Asia, east Asia, and Oceania | 0.544 | Low-middle SDI |
| Mauritania | Western Sub-Saharan Africa | Sub-Saharan Africa | 0.496 | Low-middle SDI |
| Mauritius | Southeast Asia | Southeast Asia, east Asia, and Oceania | 0.705 | High-middle SDI |
| Mexico | Central Latin America | Latin America and Caribbean | 0.649 | Middle SDI |
| Micronesia (Federated States of) | Oceania | Southeast Asia, east Asia, and Oceania | 0.580 | Low-middle SDI |
| Monaco | Western Europe | High income | 0.902 | High SDI |
| Mongolia | Central Asia | Central Europe, eastern Europe, and central Asia | 0.606 | Low-middle SDI |
| Montenegro | Central Europe | Central Europe, eastern Europe, and central Asia | 0.791 | High-middle SDI |
| Morocco | North Africa and Middle East | North Africa and Middle East | 0.548 | Low-middle SDI |
| Mozambique | Eastern Sub-Saharan Africa | Sub-Saharan Africa | 0.307 | Low SDI |
| Myanmar | Southeast Asia | Southeast Asia, east Asia, and Oceania | 0.521 | Low-middle SDI |
| Namibia | Southern Sub-Saharan Africa | Sub-Saharan Africa | 0.612 | Middle SDI |
| Nauru | Oceania | Southeast Asia, east Asia, and Oceania | 0.618 | Middle SDI |
| Nepal | South Asia | South Asia | 0.422 | Low SDI |
| Netherlands | Western Europe | High income | 0.883 | High SDI |
| New Zealand | Australasia | High income | 0.840 | High SDI |
| Nicaragua | Central Latin America | Latin America and Caribbean | 0.517 | Low-middle SDI |
| Niger | Western Sub-Saharan Africa | Sub-Saharan Africa | 0.162 | Low SDI |
| Nigeria | Western Sub-Saharan Africa | Sub-Saharan Africa | 0.515 | Low-middle SDI |
| Niue | Oceania | Southeast Asia, east Asia, and Oceania | 0.711 | High-middle SDI |
| North Macedonia | Central Europe | Central Europe, eastern Europe, and central Asia | 0.744 | High-middle SDI |
| Northern Mariana Islands | Oceania | Southeast Asia, east Asia, and Oceania | 0.771 | High-middle SDI |
| Norway | Western Europe | High income | 0.913 | High SDI |
| Oman | North Africa and Middle East | North Africa and Middle East | 0.783 | High-middle SDI |
| Pakistan | South Asia | South Asia | 0.449 | Low SDI |
| Palau | Oceania | Southeast Asia, east Asia, and Oceania | 0.738 | High-middle SDI |
| Palestine | North Africa and Middle East | North Africa and Middle East | 0.588 | Low-middle SDI |
| Panama | Central Latin America | Latin America and Caribbean | 0.686 | Middle SDI |
| Papua New Guinea | Oceania | Southeast Asia, east Asia, and Oceania | 0.394 | Low SDI |
| Paraguay | Tropical Latin America | Latin America and Caribbean | 0.638 | Middle SDI |
| Peru | Andean Latin America | Latin America and Caribbean | 0.648 | Middle SDI |
| Philippines | Southeast Asia | Southeast Asia, east Asia, and Oceania | 0.623 | Middle SDI |
| Poland | Central Europe | Central Europe, eastern Europe, and central Asia | 0.802 | High-middle SDI |
| Portugal | Western Europe | High income | 0.743 | High-middle SDI |
| Puerto Rico | Caribbean | Latin America and Caribbean | 0.814 | High SDI |
| Qatar | North Africa and Middle East | North Africa and Middle East | 0.830 | High SDI |
| Republic of Korea | High-income Asia Pacific | High income | 0.878 | High SDI |
| Republic of Moldova | Eastern Europe | Central Europe, eastern Europe, and central Asia | 0.696 | High-middle SDI |
| Romania | Central Europe | Central Europe, eastern Europe, and central Asia | 0.760 | High-middle SDI |
| Russian Federation | Eastern Europe | Central Europe, eastern Europe, and central Asia | 0.805 | High-middle SDI |
| Rwanda | Eastern Sub-Saharan Africa | Sub-Saharan Africa | 0.429 | Low SDI |
| Saint Kitts and Nevis | Caribbean | Latin America and Caribbean | 0.746 | High-middle SDI |
| Saint Lucia | Caribbean | Latin America and Caribbean | 0.670 | Middle SDI |
| Saint Vincent and the Grenadines | Caribbean | Latin America and Caribbean | 0.627 | Middle SDI |
| Samoa | Oceania | Southeast Asia, east Asia, and Oceania | 0.641 | Middle SDI |
| San Marino | Western Europe | High income | 0.884 | High SDI |
| Sao Tome and Principe | Western Sub-Saharan Africa | Sub-Saharan Africa | 0.502 | Low SDI |
| Saudi Arabia | North Africa and Middle East | North Africa and Middle East | 0.805 | High-middle SDI |
| Senegal | Western Sub-Saharan Africa | Sub-Saharan Africa | 0.389 | Low SDI |
| Serbia | Central Europe | Central Europe, eastern Europe, and central Asia | 0.767 | High-middle SDI |
| Seychelles | Southeast Asia | Southeast Asia, east Asia, and Oceania | 0.724 | High-middle SDI |
| Sierra Leone | Western Sub-Saharan Africa | Sub-Saharan Africa | 0.347 | Low SDI |
| Singapore | High-income Asia Pacific | High income | 0.861 | High SDI |
| Slovakia | Central Europe | Central Europe, eastern Europe, and central Asia | 0.812 | High SDI |
| Slovenia | Central Europe | Central Europe, eastern Europe, and central Asia | 0.840 | High SDI |
| Solomon Islands | Oceania | Southeast Asia, east Asia, and Oceania | 0.407 | Low SDI |
| Somalia | Eastern Sub-Saharan Africa | Sub-Saharan Africa | 0.081 | Low SDI |
| South Africa | Southern Sub-Saharan Africa | Sub-Saharan Africa | 0.678 | Middle SDI |
| South Sudan | Eastern Sub-Saharan Africa | Sub-Saharan Africa | 0.363 | Low SDI |
| Spain | Western Europe | High income | 0.767 | High-middle SDI |
| Sri Lanka | Southeast Asia | Southeast Asia, east Asia, and Oceania | 0.690 | High-middle SDI |
| Sudan | North Africa and Middle East | North Africa and Middle East | 0.515 | Low-middle SDI |
| Suriname | Caribbean | Latin America and Caribbean | 0.636 | Middle SDI |
| Sweden | Western Europe | High income | 0.872 | High SDI |
| Switzerland | Western Europe | High income | 0.929 | High SDI |
| Syrian Arab Republic | North Africa and Middle East | North Africa and Middle East | 0.619 | Middle SDI |
| Taiwan (Province of China) | East Asia | Southeast Asia, east Asia, and Oceania | 0.868 | High SDI |
| Tajikistan | Central Asia | Central Europe, eastern Europe, and central Asia | 0.539 | Low-middle SDI |
| Thailand | Southeast Asia | Southeast Asia, east Asia, and Oceania | 0.687 | Middle SDI |
| Timor-Leste | Southeast Asia | Southeast Asia, east Asia, and Oceania | 0.514 | Low-middle SDI |
| Togo | Western Sub-Saharan Africa | Sub-Saharan Africa | 0.417 | Low SDI |
| Tokelau | Oceania | Southeast Asia, east Asia, and Oceania | 0.626 | Middle SDI |
| Tonga | Oceania | Southeast Asia, east Asia, and Oceania | 0.636 | Middle SDI |
| Trinidad and Tobago | Caribbean | Latin America and Caribbean | 0.757 | High-middle SDI |
| Tunisia | North Africa and Middle East | North Africa and Middle East | 0.672 | Middle SDI |
| Turkey | North Africa and Middle East | North Africa and Middle East | 0.748 | High-middle SDI |
| Turkmenistan | Central Asia | Central Europe, eastern Europe, and central Asia | 0.670 | Middle SDI |
| Tuvalu | Oceania | Southeast Asia, east Asia, and Oceania | 0.589 | Low-middle SDI |
| Uganda | Eastern Sub-Saharan Africa | Sub-Saharan Africa | 0.404 | Low SDI |
| Ukraine | Eastern Europe | Central Europe, eastern Europe, and central Asia | 0.736 | High-middle SDI |
| United Arab Emirates | North Africa and Middle East | North Africa and Middle East | 0.880 | High SDI |
| United Kingdom | Western Europe | High income | 0.847 | High SDI |
| United Republic of Tanzania | Eastern Sub-Saharan Africa | Sub-Saharan Africa | 0.423 | Low SDI |
| United States of America | High-income North America | High income | 0.859 | High SDI |
| United States Virgin Islands | Caribbean | Latin America and Caribbean | 0.799 | High-middle SDI |
| Uruguay | Southern Latin America | Latin America and Caribbean | 0.697 | High-middle SDI |
| Uzbekistan | Central Asia | Central Europe, eastern Europe, and central Asia | 0.631 | Middle SDI |
| Vanuatu | Oceania | Southeast Asia, east Asia, and Oceania | 0.485 | Low-middle SDI |
| Venezuela (Bolivarian Republic of) | Central Latin America | Latin America and Caribbean | 0.607 | Low-middle SDI |
| Viet Nam | Southeast Asia | Southeast Asia, east Asia, and Oceania | 0.617 | Middle SDI |
| Yemen | North Africa and Middle East | North Africa and Middle East | 0.412 | Low SDI |
| Zambia | Eastern Sub-Saharan Africa | Sub-Saharan Africa | 0.505 | Low-middle SDI |
| Zimbabwe | Southern Sub-Saharan Africa | Sub-Saharan Africa | 0.476 | Low-middle SDI |

Note: SDI, Socio-demographic index.


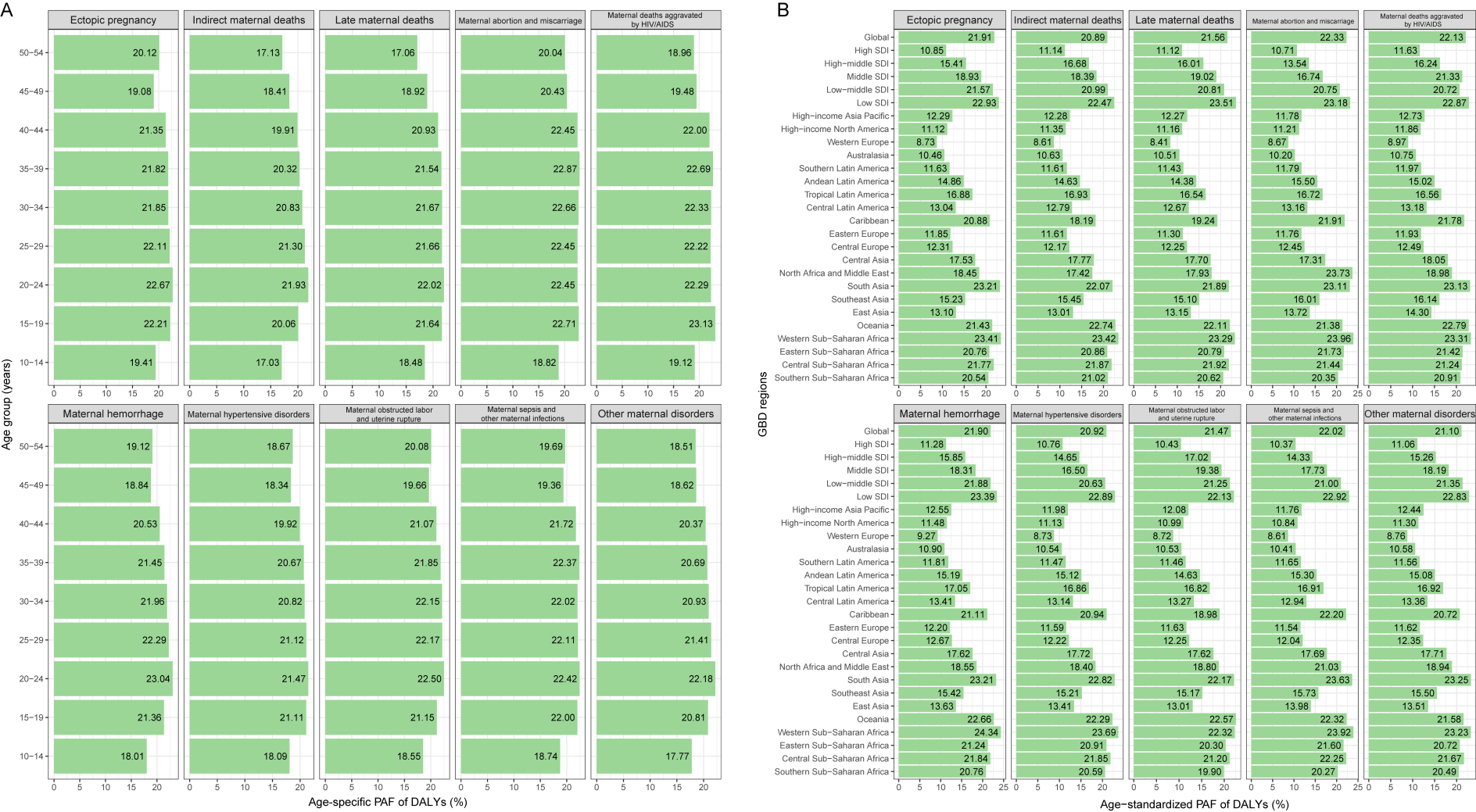


**Fig. S1** PAF of specific maternal disorders in ASDR attributable to child and maternal malnutrition by age group and by region in 2019. (A) By age group. (B) By region. ASDR, age-standardized disability-adjusted life year rate; GBD, Global Burden of Disease Study; SDI, socio-demographic index.


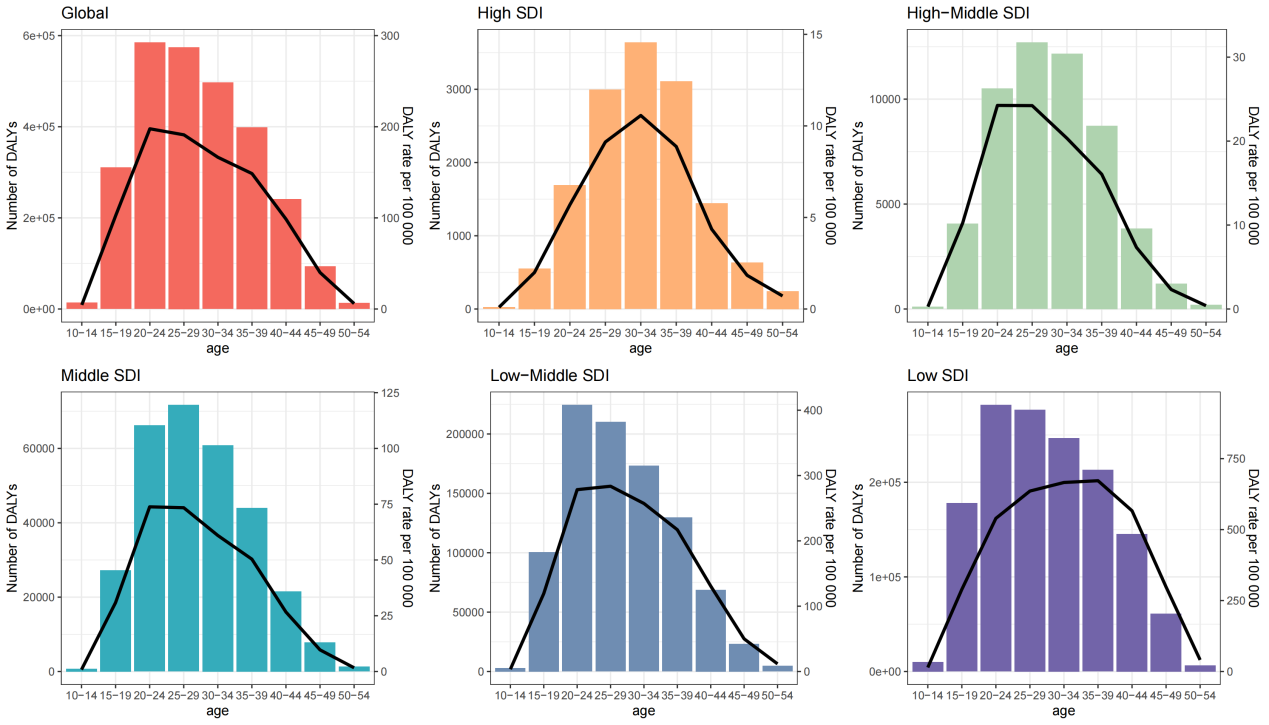


**Fig. S2** Age-specific numbers (bar plot) and rates (line plot) of DALYs of maternal disorders attributable to child and maternal malnutrition in 2019, by SDI region. DALYs, disability-adjusted life years; SDI, Socio-demographic index.


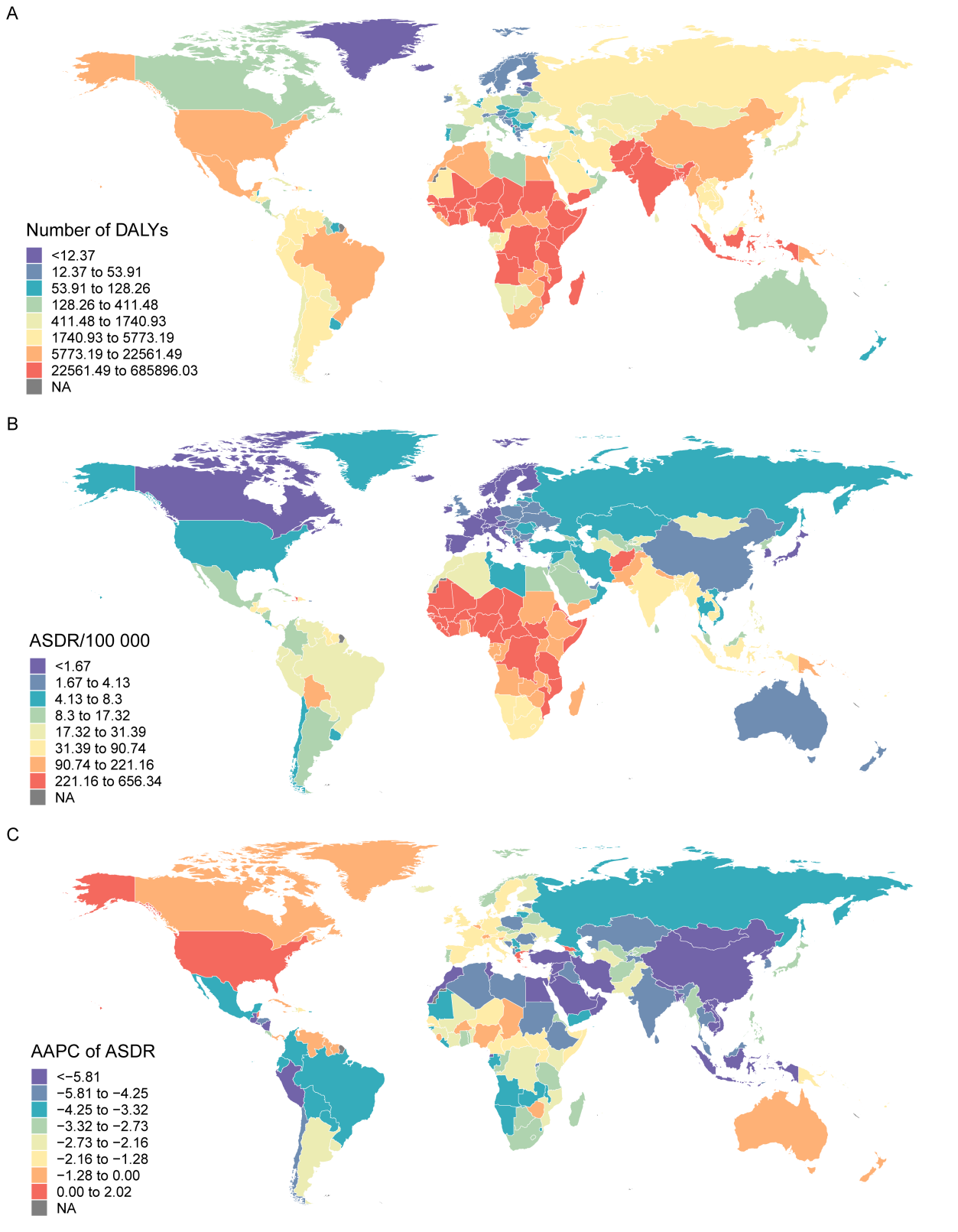


**Fig. S3** Global DALYs burden of maternal disorders attributable to child and maternal malnutrition. (A) Number of DALYs in 2019; (B) ASDR in 2019; (C) AAPC of ASDR from 1990 to 2019. ASDR, age-standardized DALYs rate; AAPC, average annual percentage change.


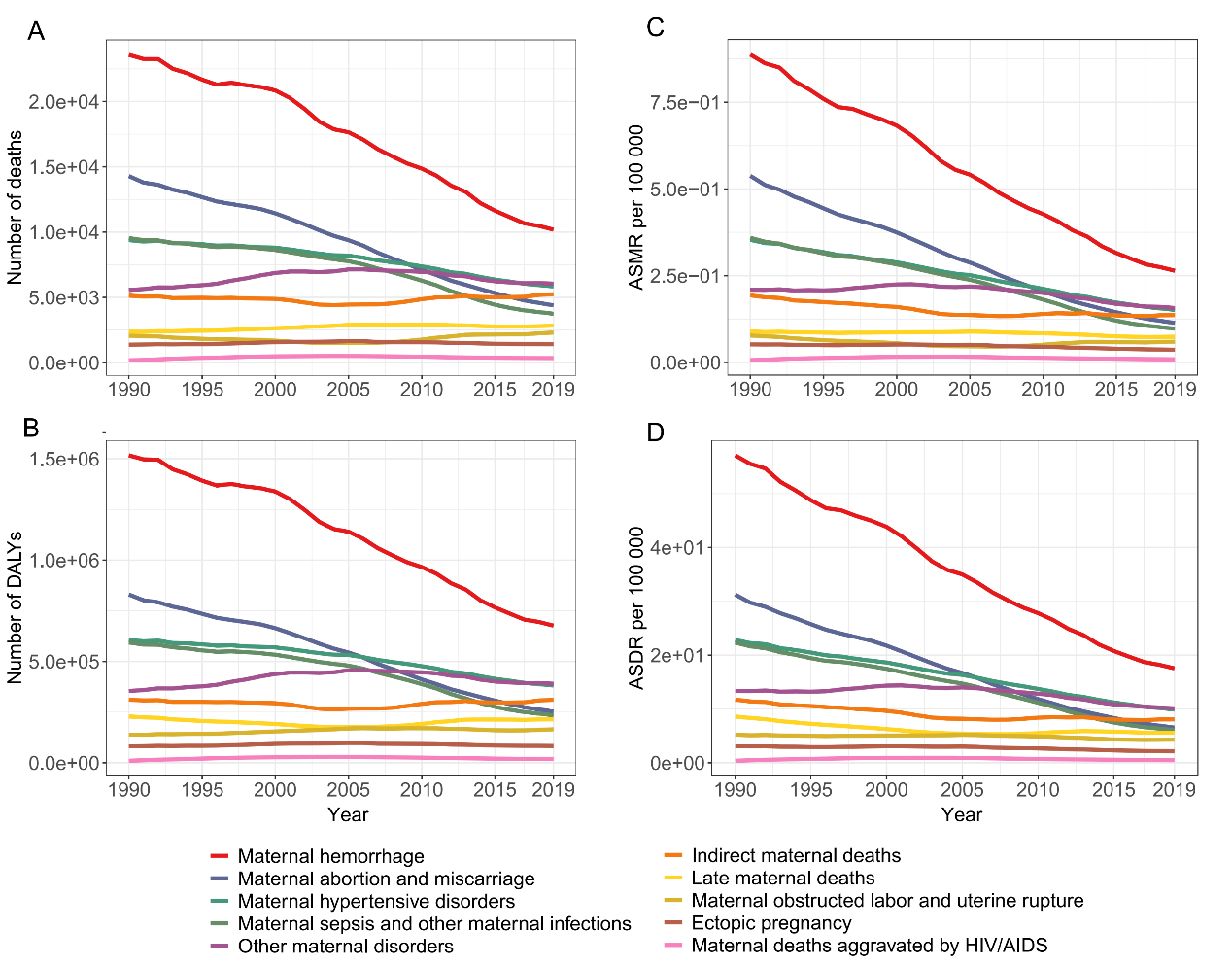


**Fig. S4** The numbers and age-standardized rates of deaths (A and B) and DALYs (C and D) of specific maternal disorders attributable to child and maternal malnutrition from 1990 to 2019. ASMR, age-standardized mortality rate; DALYs, disability-adjusted life years; ASDR, age-standardized disability-adjusted life year rate.


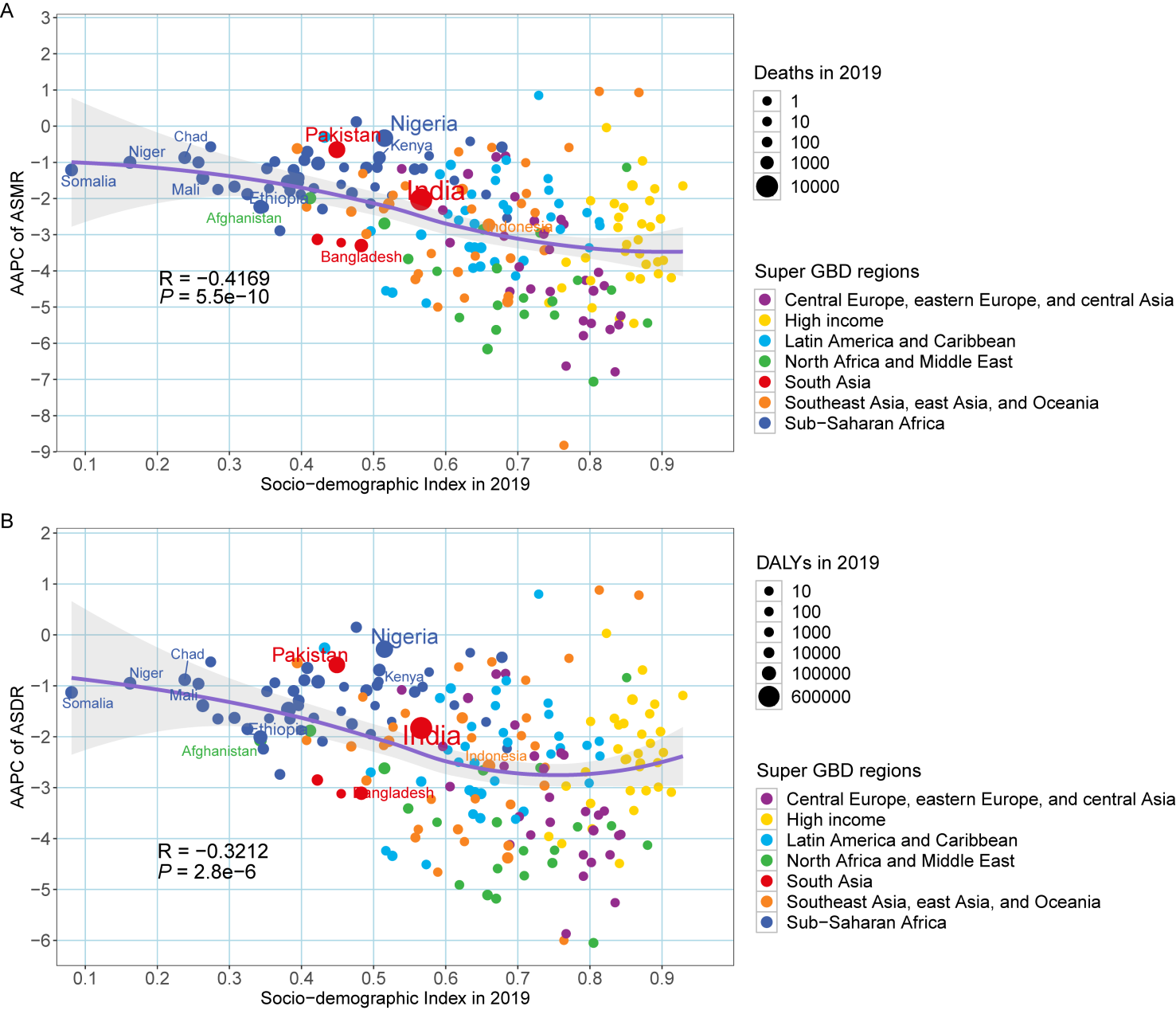


**Fig. S5** Correlations between SDI and AAPC of ASMR (A) and ASDR (B) of

maternal disorders attributable to child and maternal malnutrition in 2019. SDI,

Socio-demographic index; AAPC, average annual percentage change; ASMR,

age-standardized mortality rate; ASDR, age-standardized DALYs rate.


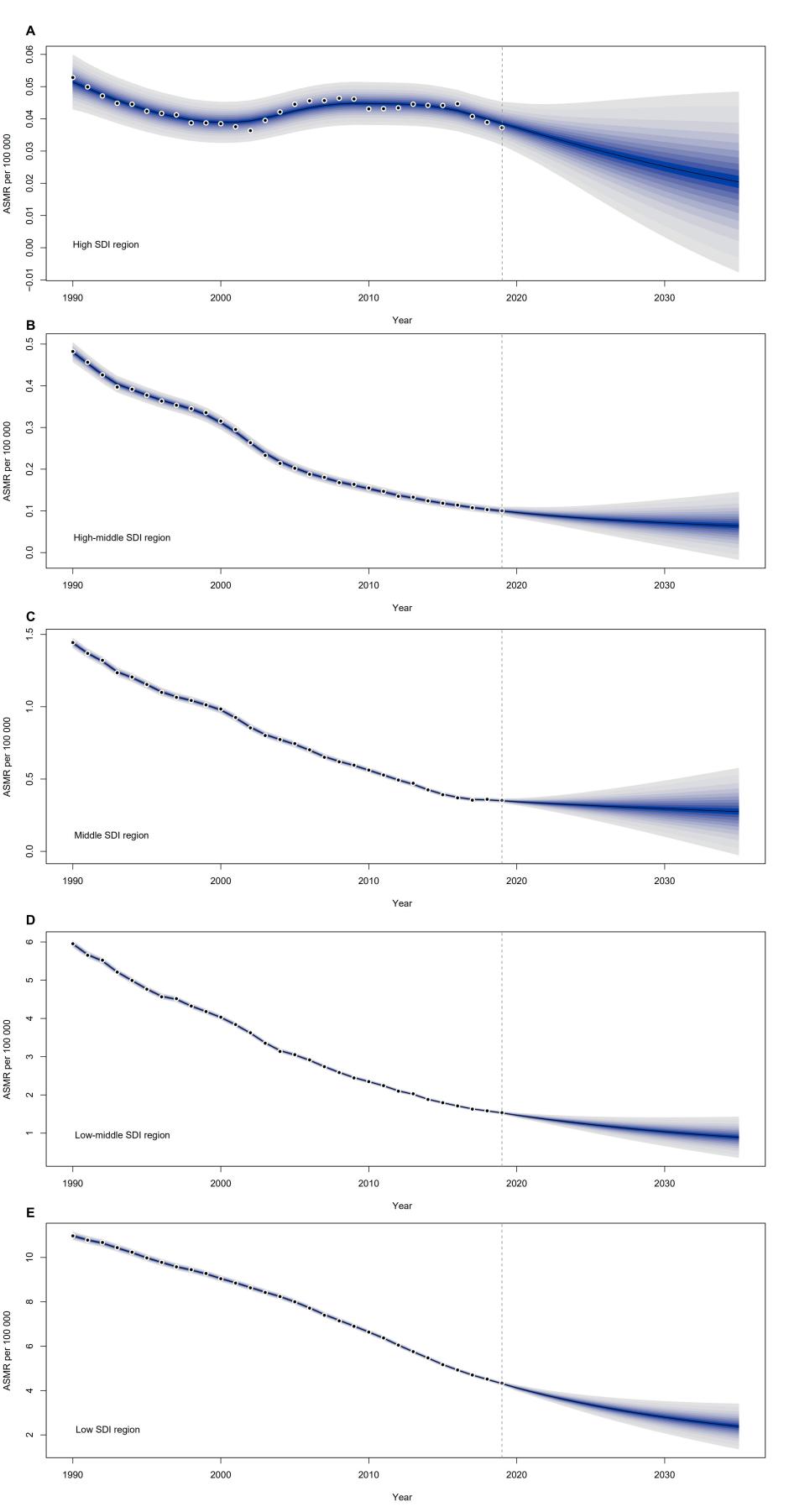


**Fig. S6** Trends in ASMR predictions for maternal disorders attributable to malnutrition, by SDI, from 1990-2035. (A) High SDI region; (B) High-middle SDI region; (C) Middle SDI region; (D) Low-middle SDI region; (E) Low SDI region. ASMR, age-standardized mortality rate; SDI, socio-demographic index.
